# Genome-Wide Analysis of the World's Sheep Breeds Reveals High Levels of Historic Mixture and Strong Recent Selection

**DOI:** 10.1371/journal.pbio.1001258

**Published:** 2012-02-07

**Authors:** James W. Kijas, Johannes A. Lenstra, Ben Hayes, Simon Boitard, Laercio R. Porto Neto, Magali San Cristobal, Bertrand Servin, Russell McCulloch, Vicki Whan, Kimberly Gietzen, Samuel Paiva, William Barendse, Elena Ciani, Herman Raadsma, John McEwan, Brian Dalrymple, other members of the International Sheep Genomics Consortium

**Affiliations:** 1 Livestock Industries, CSIRO, Brisbane, Australia; 2 Faculty of Veterinary Medicine, Utrecht University, Utrecht, the Netherlands; 3 Bioscience Research Division, Department of Primary Industries Victoria, Melbourne, Australia; 4 Laboratoire de Genetique Cellulaire, INRA, Toulouse, France; 5 Illumina Inc., San Diego, California, United States of America; 6 Genetic Resources and Biotechnology, Embrapa, Brasília, Brazil; 7 Department of General and Environmental Physiology, University of Bari, Bari, Italy; 8 Faculty of Veterinary Science, University of Sydney, Camden, Australia; 9 AgResearch, Invermay Agricultural Center, Mosgiel, New Zealand; 10 www.sheephapmap.org; The Wellcome Trust Sanger Institute, United Kingdom

## Abstract

Through their domestication and subsequent selection, sheep have been adapted to thrive in a diverse range of environments. To characterise the genetic consequence of both domestication and selection, we genotyped 49,034 SNP in 2,819 animals from a diverse collection of 74 sheep breeds. We find the majority of sheep populations contain high SNP diversity and have retained an effective population size much higher than most cattle or dog breeds, suggesting domestication occurred from a broad genetic base. Extensive haplotype sharing and generally low divergence time between breeds reveal frequent genetic exchange has occurred during the development of modern breeds. A scan of the genome for selection signals revealed 31 regions containing genes for coat pigmentation, skeletal morphology, body size, growth, and reproduction. We demonstrate the strongest selection signal has occurred in response to breeding for the absence of horns. The high density map of genetic variability provides an in-depth view of the genetic history for this important livestock species.

## Abstract 2

Genomic structure in a global collection of domesticated sheep reveals a history of artificial selection for horn loss and traits relating to pigmentation, reproduction, and body size.

## Author Summary

During the process of domestication, mankind recruited animals from the wild into a captive environment, changing their morphology, behaviour, and genetics. In the case of sheep, domestication and subsequent selection by their animal handlers over thousands of years has produced a spectrum of breeds specialised for the production of wool, milk, and meat. We sought to use this population history to search for the genes that directly underpin phenotypic variation. We collected DNA from 2,819 sheep, belonging to 74 breeds sampled from around the world, and assessed the genotype of each animal at nearly 50,000 locations across the genome. Our results show that sheep breeds have maintained high levels of genetic diversity, in contrast to other domestic animals such as dogs. We also show that particular regions of the genome contain strong evidence for accelerated change in response to artificial selection. The most prominent example was identified in response to breeding for the absence of horns, a trait now common across many modern breeds. Furthermore, we demonstrate that other genomic regions under selection in sheep contain genes controlling pigmentation, reproduction, and body size.

## Introduction

Man's earliest agricultural systems were based on the captive management of sheep and goats. The transition from hunting to animal husbandry involved human control over the reproduction, diet, and protection of animals. The process of domestication was initiated approximately 11,000 years ago in the Fertile Crescent [1]. The impact was a profound redirection of human society, as domesticated livestock and plants increased the stability of human subsistence and fuelled population growth and expansion. Domestication also reshaped the morphology, behaviour, and genetics of the animals involved, with the first consequences likely to have included changes to coat pigmentation and horn morphology. Sheep were first reared for access to meat before human mediated specialisation for wool and milk commenced ca 4,000–5,000 years ago [2]. Phenotypic radiation under selection is ongoing, resulting in a spectrum of modern breeds adapted to a diverse range of environments and exhibiting the specialised production of meat, milk, and fine wool. The last few hundred years has seen the pace of genetic gain increase dramatically through the division of animals into breeds, the implementation of quantitative genetics methodology, and the use of artificial insemination to prioritise genetically superior rams.

Patterns of genetic variation have long proven insightful for the study of domestication, breed formation, population structure, and the consequences of selection. Variation within the mitochondrial genome has documented the global dispersal of two major haplogroups in modern sheep [3],[4]. Analysis of endogenous retroviruses suggests the development of breeds has occurred in multiple waves, where primitive breeds have been displaced by populations which display improved production traits [2]. Investigations into the genetic relationship between populations have primarily relied on a modest collections of autosomal microsatellites [5]–[7], Y chromosomal markers [8], or SNP [9]. To date, the majority of populations tested have been European-derived breeds. This prompted assembly of the global sheep diversity panel, which contains animals from 74 diverse breeds sampled from Asia, Africa, South-West Asia (the Middle East), the Caribbean, North and South America, Europe, and Australasia. Our goal in assembling this animal resource was 2-fold. Firstly, we sought to examine levels and gradients of genetic diversity linking global sheep populations to better understand the genetic composition and history of sheep. We therefore genotyped all of the animals in the global diversity panel using the *ovine* SNP50 Beadchip, an array consisting of approximately 50,000 evenly spaced SNP. We present the relationship between breeds in terms of divergence time, estimated from the extent of haplotype sharing. Secondly, we sought to characterise the genetic legacy that selection and adaptation have imparted on the sheep genome. By performing a genome-wide scan for the signatures of selection, 31 genomic regions were identified that contain genes for coat pigmentation, skeletal morphology, body size, growth, and reproduction. By combining the collection of a global sample of ovine breeds with the ability to interrogate 50,000 genetic loci, the results provide unprecedented insight into the phylogeographic structure of sheep populations and the results of centuries of breeding practices.

## Results

### High Levels of Polymorphism and Genetic Diversity

**Figure 1:**
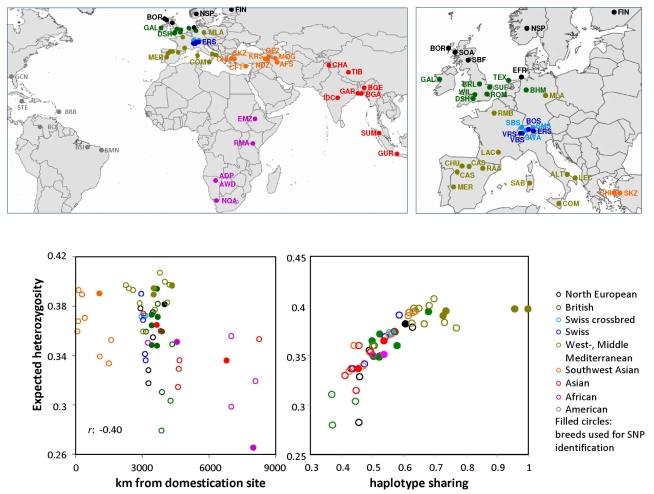
Geographic origin of breed development and diversity. Breeds were genotyped from the Americas, Africa, Asia, and the domestication centre in present-day Iran and Turkey (referred to throughout as South-West Asia). The majority of breeds genotyped were developed in Europe (given in detail at right). Breed names and their abbreviations are given in Table S1. Marker heterozygosity within each breed compared against increasing physical distance from the domestication centre. Breeds used during SNP discovery are shown using filled circles. Haplotype sharing at 25–50 Kb between Merinos and other breeds (Figure S8) was plotted against heterozygosity to reveal a major influence of Merino admixture on the genetic diversity of European breeds. Breed-specific values for expected heterozygosity and haplotype sharing are given in Table S4 to allow identification of populations with outlier values.

Analysis of genetic variation was performed for 2,819 animals in the global sheep diversity panel. Breeds were sampled from each continent across the species range (Figure 1), including six breeds from both Africa and America, seven from South-West Asia (the Middle East), eight from Asia, and the rest from northern, north-western, central, and southern and south-western Europe (Table S1 lists the breed and their geographic origin). All animals were genotyped using the *ovine* SNP50 Beadchip, an array consisting of SNP derived from three separate sequencing experiments (Roche 454, Illumina GA and Sanger sequencing; Table S2). A series of quality control filters were applied to identify 49,034 SNP used in subsequent analysis (Table S3). Levels of SNP polymorphism were generally high, with greater than 90% of loci displaying polymorphism within the majority of breeds (Table S4). The distribution of minor allele frequency (MAF) differed between population groups chosen to reflect the geographic origin of breed development. African and Asian breeds had an excess of low MAF SNP (<0.1) compared to European-derived populations. This partly reflects ascertainment bias in SNP discovery, as the same analysis conducted using SNP discovered without use of African or Asian sheep (454 SNP; Figure S1, Table S2) shows a more pronounced excess compared with SNP discovered using a broad genetic base (Illumina GA SNP). To examine diversity on a global scale, we calculated observed heterozygosity (*H*e) within breeds and between regions (Table S4). Allele frequency-dependent diversity estimates such as *H*e are sensitive to ascertainment bias, prompting the removal of SNP in high LD, which acts to counter the effect of the bias and generate meaningful comparisons between populations [10]. Applied here, breed rankings based on *H*e were generally stable following LD-based pruning and when calculated using SNP sets ascertained using different methods (Figure S2). Following LD-based correction, animals from Southern and Mediterranean Europe displayed the highest heterozygosity (Figure S2). This likely reflects the first migrations of Neolithic communities and their animals, following the Mediterranean as a sea route into Europe [11]–[13]. Relative levels of genetic diversity are expected to decrease with increasing distance from the domestication centre. For sheep, breed heterozygosity revealed only a weak association with increasing physical distance (Figure 1B, *r* = −0.40). This appears much less pronounced in sheep compared with human migration out of Africa [14]. One likely explanation is the widespread use of Merino sires across Europe that commenced after the Middle Ages. The result is extensive haplotype sharing between Merinos and other breeds (Figure 1C). Generally high SNP diversity in sheep was accompanied by many breeds displaying high current effective population size (*N*e, Table S4). Compared with domestic cattle where the majority of breeds have a current *N*e of 150 or less [15], estimates here revealed 25 breeds have *N*e exceeding 500 and only two sheep populations showed evidence of a comparatively narrow genetic base (*N*e<150).

### Relatedness Between Animals and Evidence for High Levels of Admixture

**Figure 2:**
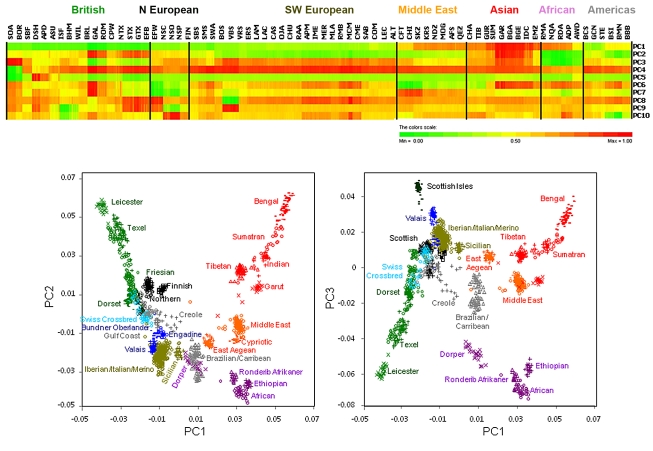
Population structure within the global sheep diversity panel. Principal component (PC) analysis of genetic distance was performed using a subset of 20,279 SNP identified by LD-based SNP pruning. Heat strips for each of the first 10 PCs are shown for 74 breeds (top panel). The PC value for each animal was normalised to range from 0 to 1 and visualised as a colour spectrum from green (0) to red (1). Plots for PC1 and 2 (bottom left) and PC1 and 3 (bottom right) each revealed the clustering of 1,612 animals selected to balance the number of animals across breeds. Individuals are colour coded to represent their geographical origin.

Global patterns of genetic structure were inferred by principal components analysis (PCA, Figure 2). The analysis ignores breed membership but revealed clear structure as animals from the same breed clustered together. As demonstrated in human and other livestock species such as cattle [15]–[17], the combination of PC1, PC2, and PC3 separated individuals according to their geographic origin. The largest PC (2.98% of total variation) positioned European sheep apart from African, Asian, and South-West Asian animals. The second PC (1.44%) separated European-derived animals from those developed in Africa and Asia animals. PC3 (1.19%) identified admixed populations such as the African Dorper and breeds developed in South America and the Caribbean were positioned away from other clusters. It also resolved two primitive and geographically isolated Scottish breeds (Soay and Boreray) as outliers from all other animals [2],[6],[9]. PC4 (1.09%) separated British Dorset types (DSH, APD, and ASU) from other European derived breeds and PC7 identified the Valais breeds as genetically distinct. Additional PCs reveal the divergence of single or a few related breeds (refer to the heatmap in Figure 2). To explore in detail the relatedness between European animals, analysis was performed separately for Mediterranean and northern-derived breeds (Figure S3). Even closely related populations such as Irish and Australia Suffolk had non-overlapping clusters, confirming the dataset provides an extremely high resolution view of population divergence. This power of resolution results from the large number of markers used, as a pilot study using only 1,315 SNP failed to distinguish closely related European-derived breeds [9]. Model-based clustering partitioned the genome of each animal into a predefined number of components (*K*) [18]. For unsupervised clustering assuming two ancestral populations (*K* = 2), a clear division was observed between Northern European and Asian breeds (Figure S4), corresponding to PC1. Clusters were reproducible up to *K* = 9 and grouped individuals according to their geographic origin in the same way as for PCA (Figure 2). The 20 largest PCs accounted for only 16% of the total variation (Figure S5), consistent with reports suggesting sheep have a weak population structure [3],[9]. To evaluate if this was accompanied by high levels of haplotype sharing between breeds, the extent of LD was characterised by the signed *r* statistic between SNP pairs at different lengths (e.g., [19]). For SNP pairs separated by 10 kb or less, a high degree of conservation of LD phase was observed between all breeds (Figure S7). Given that LD at short haplotype lengths reflects population history many generations ago [20],[21], this also supports a common ancestral origin of all domestic breeds of sheep. The result is in contrast to cattle, where two distinct groups emerge from a similar analysis, even at haplotype lengths of 0–10 kb, reflecting the *Bos taurus taurus* and *Bos taurus indicus* sub-species and their separate domestication events [15]. To determine if our LD-based estimates of haplotype sharing and effective population size were influenced by strong admixture, simulation was performed using a mutation drift model [22] and populations designed to mimic HapMap sheep breeds. This revealed admixture did affect inferred *N*e, however the impact was minimal outside of the period in which the admixture took place (Figure S6).

### Phylogenetic Relationship Between Breeds

**Figure 3:**
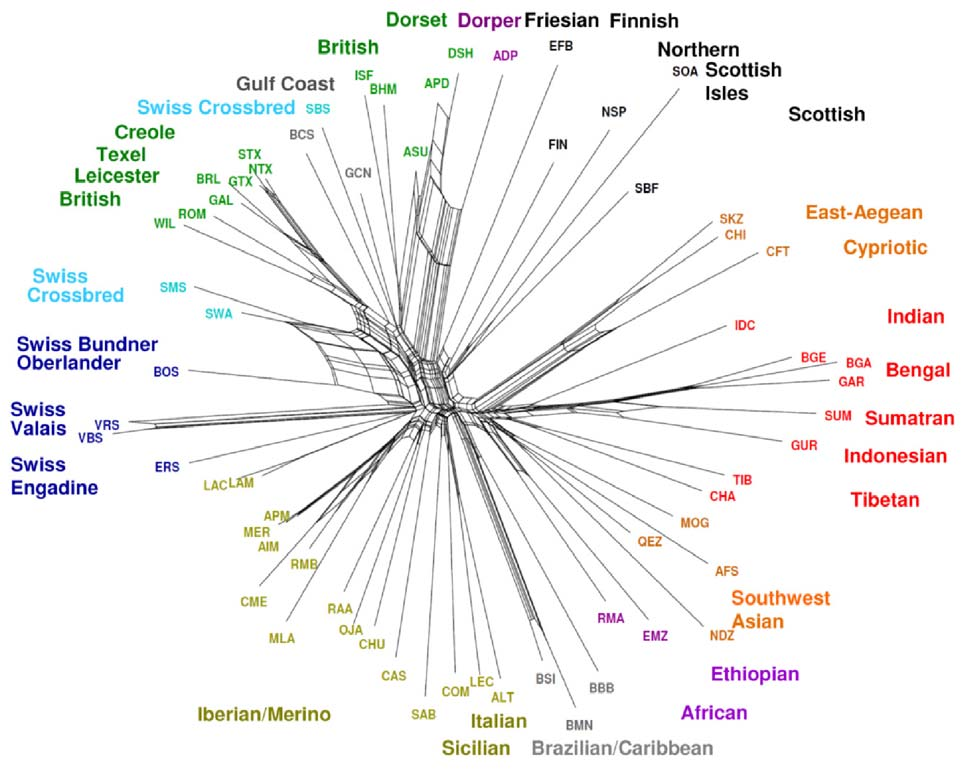
Relationship between breeds based on divergence time. The divergence time between breeds (in generations) estimated using LD was used to draw a NeighborNet graph. Reticulations towards the extremity of each graph indicate increasing genetic relatedness between populations. The divergence times are visualised as a heatmap in Figure S10.

**Figure 4:**
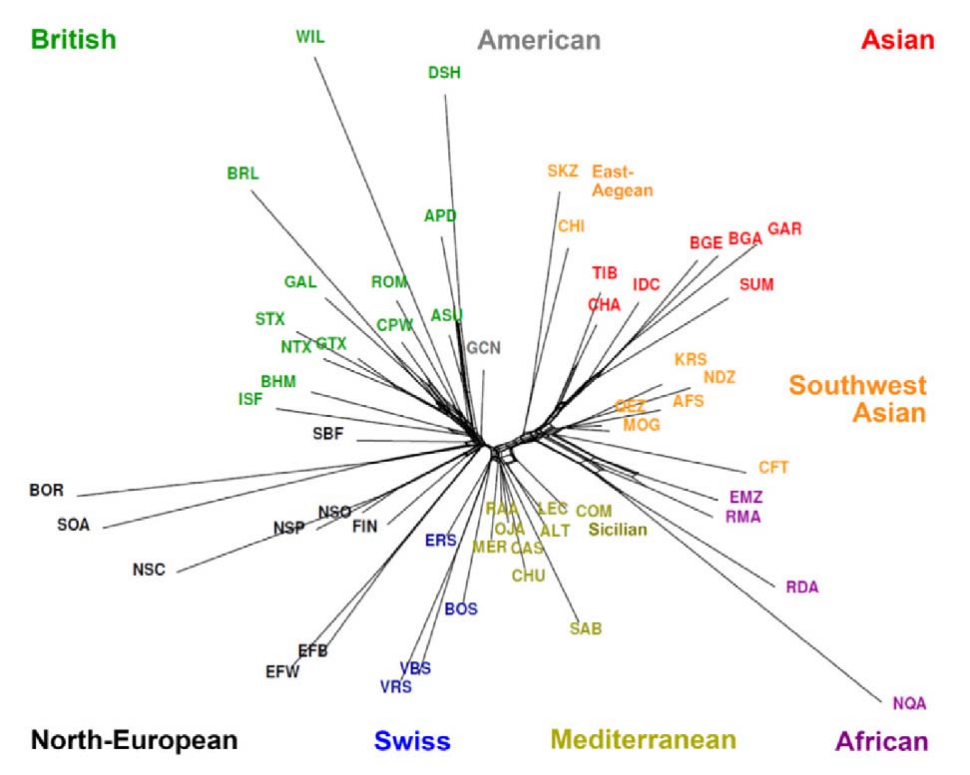
Relationship between breeds based on Reynolds distance. An allele frequency-dependent distance metric (Reynolds) was used to construct a NeighborNet graph relating breeds. As for Figure 3, reticulations towards the extremity of each graph indicate increasing genetic relatedness between populations.

The relationship between breeds was examined using two distance metrics. Firstly, the divergence time separating all breed pairs was estimated from LD and haplotype sharing using the methods of (Figures S7, S8, S9, S10) [19]. Divergence time (in generations) revealed a strong correspondence with known population history for recently separated breed pairs. For example, breeds established within the last 100 years (e.g., Poll Dorset and Poll Merino) had the shortest divergence time (<80 generations). Breeds with longer history, such as American Rambouillet, had divergence from Merino estimated at 160–240, which matches with their export from Spain to America starting in the late 1800s. The deepest divergence was estimated at only 800 generations, which appears to be an underestimate likely reflecting the influence of admixture. Divergence times between all breeds were explored as a NeighborNet graph that had branches of approximately equal length, suggesting the approach is robust to differences in genetic drift and effective population size between populations (Figure 3). NeighborNet graphs allow for reticulation as a consequence of relatedness and mixed breed origin, and the topology of the graph reproduced both the geographic groups and relationships obtained by PCA. Reticulations were observed toward the extremity of the graph for breed pairs that clustered together in PCA (e.g., Dorset Horn and Australia Poll Dorset). Conversely breeds identified as outliers by PCA such as the Soay had branches that originated from the centre of the graph. The second distance metric, Reynold's distance, relies on allele frequency differences, and branch lengths were highly variable (Figure 4). To test for the impact of ascertainment bias in SNP selection, we compared graphs generated using different SNP sets. In each case, the graphs had highly similar topology, which argues against a major influence of bias during SNP discovery (Figure S11). Short branches were observed for Spanish, Italian, and Iranian breeds with a high heterozygosity, while long branches were found for isolated populations containing small effective population size. Omitting the crossbred populations resulted in a remarkable demarcation of the geographic clusters. The topology of the graph suggests a major migration route along an axis that runs from South-West Asia to the Mediterranean region and via central Europe to Britain and the Nordic regions. Testing of additional breeds will be required to assess if migration was strongly influenced by a Danubian colonisation route.

### Signals of Selection

**Figure 5:**
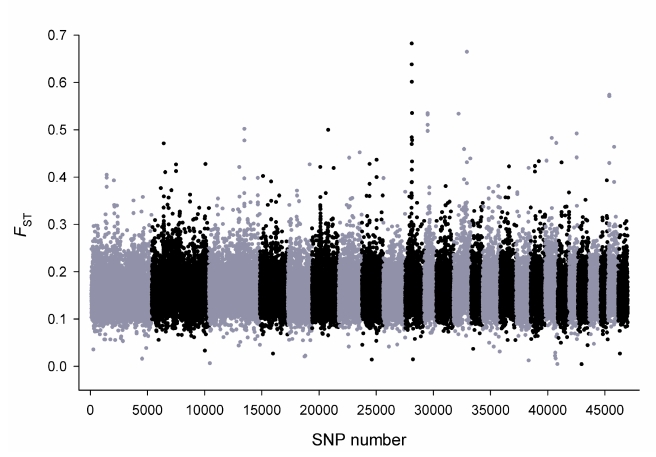
Genome-wide distribution of global *F*_ST_. The amount of differentiation, measured as *F*_ST_, was estimated within each breed by comparison to all other breeds. Global *F*_ST_ is the average for each SNP across all 74 HapMap breeds, meaning common signals present in multiple breeds are preferentially detected. SNP were ordered in genomic order with OAR1 at left. The highest peak is on OAR10.

**Figure 6:**
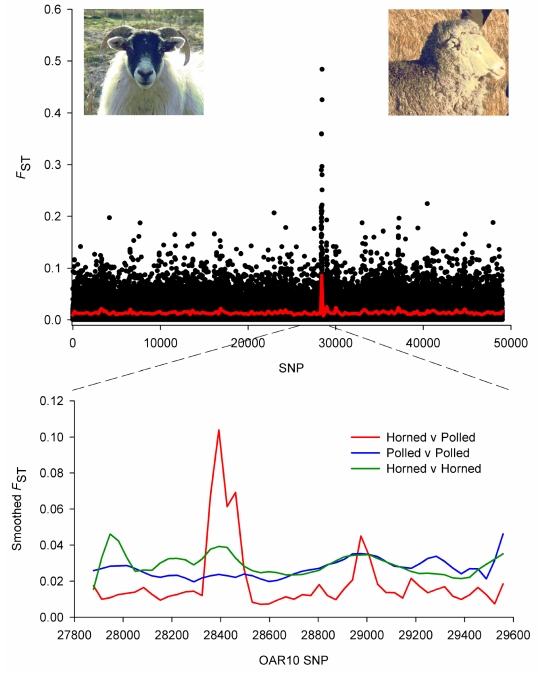
Selection for sheep without horns (poll). Animals from two breeds with horns (Dorset Horn and Merino) were pooled and compared with two polled breeds (Poll Dorset and Poll Merino). Pairwise *F*_ST_ was calculated between the two groups of animals for all 49,034 SNP, before smoothed values were plotted in order across the genome (top panel). A strong selection signal was observed on Chromosome 10 (SNP number 27,878–29,558 with the signal peak at SNP *OAR10_29546872*). Pairwise *F*_ST_ was also calculated between horned breeds (green line) or between polled breeds (blue line) before the smoothed values were plotted across Chromosome 10 (bottom panel). The peak was only observed where horned breeds were compared with polled breeds, verifying that the signal relates to the long-standing husbandry practise of selecting animals for the absence of horns.

Animal husbandry and directed mating have been used to successfully adapt sheep to a diverse range of environments and to the specialised production. Selection is predicted to alter allele frequencies within the target population for both functional mutation(s) and their neighbouring SNP. Global *F*_ST_ was calculated, which measures differentiation within each breed versus all other breeds and detects both positive and balancing selection. The genome-wide distribution of global *F*_ST_ for 49,034 SNP revealed the highest selection signal was detected on Chromosome 10 (Figure 5). The highest ranked SNP (*OAR10_29511510*; *F*_ST_ = 0.682) was located at Mb position 29.54 near the Relaxin/insulin-like family peptide receptor 2 (*RXFP2*), which was recently linked with the absence of horns (poll) in sheep [23] and displayed strong evidence for selection in cattle [24]. This prompted calculation of pairwise *F*_ST_ between breeds defined as either being horned or polled. This recapitulated a single strong and striking selection signal at *RXFP2* (Figure 6). Importantly, the *F*_ST_ signal was absent when polled breeds or horned were compared with each other.

A total of 31 genomic regions contained the top 0.1% of markers ranked using global *F*_ST_ (47 SNP, Table 1). This implicated 17.85 Mb of sequence containing 181 genes as being under selection. The exact target of selection was difficult to identify as six genes, on average, were present within each genomic region. Gene ontology (GO) terms associated with the 181 genes were evaluated for evidence of functional enrichment against a background set of 11,098 genes physically tagged by the *ovine* SNP50 Beadchip (Table S5). This revealed enrichment for GO terms associated with regulation of bone remodelling (*p* = 5.5×10^−5^) and bone resorption (*p* = 4.0×10^−5^). Given it is unlikely all 181 genes have undergone selection but each contributed to the GO analysis, caution is required during interpretation. Nonetheless, the content of the differentiated regions strongly suggests enrichment for genes under selection given their roles in pigmentation, body size, reproduction, animal production, and domestication. Selection for specialised coat pigmentation represents breed-defining characteristics across domestic animals including sheep. Selection signals were detected spanning *KIT*, *ASIP*, and *MITF* (regions 8, 19, and 26 on OAR 6, 13, and 19, respectively, Table 1). *KIT* and *MITF* interact during melanocyte development and account for pigmentation phenotypes in pigs and cattle [25],[26], while duplication of *ASIP* in sheep controls a series of alleles for black and white coat colour [27]. Global *F*_ST_ peaks spanned *NPR2*, *HMGA2*, and *BMP2*, which are each involved in skeletal morphology and body size (regions 1, 5, and 18 on OAR 1, 5, and 18, respectively, Table 1). *HMGA2* is of particular interest as it was recently shown to be under selection in dogs with divergent stature [28],[29]. Positive selection was detected surrounding two genes known to regulate growth and reproduction (*PRLR* on OAR6l; *TSHR* on OAR 7; Table 1). Prolactin receptor (*PRLP*) is a key regulator of mammalian reproduction that is critical for the onset of lactation and is associated with milk traits in dairy cattle [30]. In addition, a very strong selection sweep surrounds the thyroid stimulating hormone receptor (*TSHR*) in chicken, which given its pivotal role in metabolic regulation and the control of reproduction, was postulated to be a domestication gene [31]. Finally, an *F*_ST_ peak on Chromosome 6 spanned the *FGF5* gene, recently shown to contain mutations in dog responsible for variation in hair type [32]. Each putative gene target for selection is recorded in Table 1, however this does not include examples where the 31 regions intersect with previous findings arising from QTL that have not been resolved to identify individual genes. One example is Mb position 6.8–7.2 on OAR 25, which contains QTL for wool production and quality in a number of breeds [33],[34]. The location of all 31 regions were compared to selection signals identified within the cattle genome [15],[24],[35]–[40]. Eleven of the 31 genomic regions identified here appear to be under selection in cattle, suggesting genes such as *KIT*, *FGF5*, *MITF*, and *RXFP2* are targets for selection across multiple mammalian lineages (Table S6).

**Table 1:** Regions under selection in the sheep genome.

Region	Chr	Position (Mb)	Peak SNP (*F*_ST_)	Top SNP	Genes	Candidates
1	2	55.25–56.98	*s20468* (0.471)	1	47	NPR2
2	2	111.72–112.11	*s29378* (0.426)	2	5	
3	2	246.07–246.46	*s01865* (0.427)	1	8	CHRNG
4	3	141.36–141.61	*OAR3_141586525* (0.421)	1	14	HOX gene cluster
5	3	164.38–165.45	*OAR3_165009241* (0.502)	2	4	HMGA2, MSRB3
6	5	105.87–105.93	*s36709* (0.427)	1	1	
7	6	40.21–42.53	*OAR6_40277406* (0.421)	1	18	ABCG2, PDK2
8	6	76.38–76.86	*OAR6_76473607* (0.499)	1	3	KIT
9	6	103.41–103.99	*s21552* (0.419)	1	7	FGF5
10	7	49.12–49.22	*s69881* (0.441)	1	1	
11	7	97.3–97.72	*OAR7_97378846* (0.452)	1	5	TSHR
12	8	34.2–35.35	*s20065* (0.428)	1	6	BVES
13	8	67.47–67.84	*OAR8_67529714* (0.436)	1	3	
14	10	29.27–29.98	*OAR10_29511510* (0.682)	6	3	RXFP2
15	10	30.6–30.79	*s68983* (0.535)	3	2	
16	11	18.57–19	*OAR11_18701428* (0.535)	4	4	NF1
17	13	25.27–25.57	*s71551* (0.534)	1	1	
18	13	51.54–52.64	*OAR13_51852034* (0.459)	1	1	BMP2
19	13	67.07–68.54	*s51670* (0.664)	2	16	ASIP
20	13	84.89–84.98	*s54638* (0.439)	1	1	NFATC2
21	16	41.74–42.43	*OAR16_41943180* (0.422)	1	3	PRLR
22	17	64.75–64.89	*s41543* (0.419)	1	0	
23	18	19.65–20.23	*s31152* (0.423)	1	3	ABHD2
24	18	40.36–40.58	*s45597* (0.433)	1	2	FOXG1
25	19	7.41–7.6	*s38567* (0.434)	1	3	GLB1
26	19	33.09–33.61	*OAR19_33278780* (0.483)	1	1	MITF
27	19	55.99–56.25	*s18532* (0.472)	1	2	CCR2
28	20	18.06–18.36	*OAR20_18263165* (0.43)	1	4	VEGFA
29	21	45.19–45.58	*s11631* (0.492)	2	6	OR2AG1
30	25	6.82–7.61	*s03686* (0.573)	3	4	
31	25	29.03–29.19	*s10489* (0.464)	1	3	CDH23

A total of 31 genomic regions contained the top 0.1% of SNP ranked using global *F*_ST_ (47 SNP). The top 5% of SNP were used to define the boundaries of each region using a stepping approach (see Materials and Methods). The number of genes and number of top SNP (0.1%) are given for each region along with candidates for selection.

**Figure 7:**
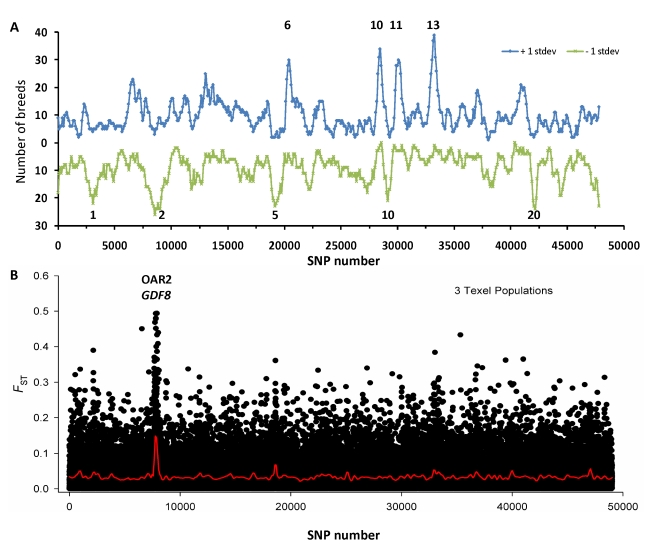
Common selection signals. The number of breeds that showed divergent selection is shown as a function of genomic position (A). Selection peaks were defined as regions with smoothed *F*_ST_ in excess of one standard deviation either above (positive selection; blue line) or below the genome-wide average (balancing selection; green line). Four regions were identified with shared positive selection peaks in 30 or more breeds (the chromosomal number is given above each peak). Similarly, five peaks were identified where 20 or more populations shared balancing selection, including the MHC region on OAR 20. One signal was common to each of three separate populations of Texel (B). Pairwise *F*_ST_ was calculated between Texel and all other animals, which revealed a strong selection on sheep Chromosome 2 above the *GDF8* gene, which underpins a breed defining phenotype.

To search for selection observed across multiple breeds, the number of populations that displayed divergence was plotted across the genome. This revealed peaks where selection was shared across breeds, and troughs where signals were absent or unique to only a small number of breeds. Four regions were detected with positive selection shared across 30 or more breeds, while five different regions were observed with shared balancing selection. The strongest balancing selection signal was observed for the MHC region on sheep Chromosome 20 (Figure 7), a result previously observed in other species including cattle [37]. Conversely, some selection signals were breed specific. The global sheep diversity panel contained three geographically separate samples of the Texel, a meat sheep known for its growth and muscling (Table S1). When Texels were grouped and compared against all other animals, a strong peak was detected on Chromosome 2 (Figure 7). The peak spans *GDF8*, a gene known to carry a mutation in Texel responsible for muscle hypertrophy [41].

## Discussion

Access to patterns of SNP diversity within a global sample of domestic sheep was used to examine the population history of a species amongst the first to be domesticated by man. Our analysis revealed this domestication process must have involved a genetically broad sampling of wild stock. Approximately 75% of modern sheep breeds have retained an effective population size in excess of 300, higher than cattle and much higher than most breeds of dog. This suggests a highly heterogeneous pre-domestication population was recruited, and the genetic bottleneck which took place was not as severe during the development of sheep as for some other animal domesticates. It is also possible that cross-breeding with wild populations persisted following the initial domestication events to generate the diversity observed. Surveys of ovine mtDNA variability support a broad genetic base during domestication, with at least five lineages identified within modern breeds that diverged well before domestication approximately 11,000 years ago [4],[11],[42],[43]. Three aspects of the SNP diversity documented in this study indicated high levels of gene flow have occurred between populations following domestication. First, a high degree of conservation in LD phase and haplotype sharing across short chromosomal distances was detected amongst almost all breeds independent of geographic origin. Secondly, we did not detect a strong association between genetic diversity and physical distance from the domestication centre, and thirdly, the proportion of variation explained by principal component analysis suggests a weak global population structure. High gene flow and introgression between breeds has been postulated previously, based on the phylogeographic distribution of mtDNA lineages [3],[4]. In addition, human-mediated transportation of sheep is well documented including the export of wool sheep from Italy during the Roman period and use of British sires on the European continent from the early Middle Ages onwards [44]–[46]. What remained unclear until now, however, was the extent of admixture that accompanied these sheep transportations and the high diversity this has left within many breeds.

Inspection of a much larger number of SNP than in previous studies [9] allowed PCA and model-based clustering to successfully detect a clear phylogeographic pattern within the breeds genotyped. At a global scale, clear genetic divisions were detected separating European, Asian, and Africa sheep. This division likely reflects variation between the populations that participated in the earliest migrations outwards from the domestication centre. At the breed level, isolated populations were identified as outliers in PCA with low *N*_e_ (e.g., Soay, Wiltshire Horn, and Macarthur Merino). Conversely, sheep from the Americas (Brazil and the Caribbean) had high *N*_e_ and clustered separately from European, African, or Asian populations. Decomposing the genome into two or more components (*K*<2; Figure S4) revealed a genetic origin for Caribbean breeds in common with African animals mixed with those of Mediterranean Europe. Similar results have been observed for New World Creole cattle [24]. This likely reflects the transportation of animals during the migration of enslaved West Africans bought to the Caribbean as slave labourers starting in the 1500s and the introduction of sheep by European colonialists.

The observed patterns of genetic variation used to make inferences about population history can be explained by neutral fluctuations and the action of genetic drift. Not all loci tested in this experiment, however, appeared neutral as clear evidence was obtained for accelerated divergence in response to selection. A genome-wide scan for differentiation using global *F*_ST_ revealed 31 chromosomal regions with evidence for selection. It is important to recognise that genome scans such as this, even when conducted using a meaningfully large number of loci and animals, have several limitations. Foremost amongst these is that the identification of SNP displaying outlier behaviour is not, in itself, proof that selection has taken place. Where convincing signals are detected, it can be difficult to clearly identify the target of selection within a region, and even more difficult to establish the link between selection and its morphological consequence. In this study the strongest selection signal was identified immediately adjacent to *RXFP2*, a gene involved in reduced bone mass and sexual maturation [47],[48]. Strong evidence supports that *RXFP2* was targeted by breeding for the removal of horns, likely to be one of the oldest morphological modifications that accompanied domestication [49],[50]. The gene underpins QTL for horn morphology [23] and the selection signal was reconstituted only when comparing horned with polled populations. Taken together the results represent a rare example where selection has been detected and demonstrated to have occurred in response to a clearly identified human-mediated breeding objective. Given the long-standing nature of the selection, it was surprising it gave rise to the strongest selection signal. Our interpretation is that this reflects the widespread frequency of polled animals across a large number of breeds, as this assists in generating extreme *F*_ST_ when calculated across all breeds. Conversely, strong selection at a locus that is private to only one or two breeds is not reflected using the global *F*_ST_ metric. Selection surrounding *Myostatin* in Texel illustrates this clearly, as a strong signal is revealed when Texels are compared with all other breeds, but it is absent from the 31 regions identified using the full dataset (Figure 7 and Table 1). Analysis was performed to search for selection signatures common to more than one domesticated species. It seems reasonable to expect common signals may exist, given some breeding goals are constant across livestock species. One example is man's desire to breed animals that display consistent pigmentation type within breeds. It follows that key pigmentation genes may show evidence for selection in more than one species, and indeed that is what was detected here for genes such as *MITF* and *KIT* (Table S6).

In summary, the phenotypic variability and population history of domestic animals make them an appealing model to study the consequences of selection. This promise is being realised through the recent availability of meaningfully large collections of SNP. Applied here, patterns of diversity were examined to systematically identify genomic regions in sheep that have undergone accelerated change in response to selection. Identification of the adaptive alleles within each genomic region remains a challenge. If resolved, the outcome will be knowledge describing the functional variants that characterise differences between breeds. The analysis of genomic polymorphism conducted here carries practical consequences. With the division of animals into breeds during the last few hundred years, animal breeding has witnessed a dramatic change. Most recently, the identification of superior rams and their disproportionate genetic contribution via artificial insemination has lifted the pace of genetic gain for production traits. The population-level consequence is a dramatic reduction in effective population size, which is best illustrated for cattle where the sharp decline in *N*e already threatens breed viability [15]. The finding here that the majority of breeds have retained a high genetic diversity and effective population size implies that selection response for wool, meat, adaptation, and welfare traits may be expected to continue.

## Materials and Methods

### Animal Material, Genotyping, and SNP Quality Control

The number of animals per population and geographic origin of breed development is given in Table S1. Individuals were collected from multiple flocks to capture a representative sample of within-breed genetic diversity. Beadchip array manufacture and genotyping was performed by Illumina (San Diego, CA) before raw signal intensities were converted into genotype calls using the Genome Studio software. SNP that failed any of the five following criteria were removed: (1) markers with <0.99 call rate; (2) markers identified during clustering as having atypical X-clustering, evidence for a nearby polymorphism, compression, intensity values only, or evidence of a deletion; (3) SNP with minor allele frequency equal to zero; (4) SNP with discordant genotypes identified by comparison of 10 animals genotyped independently at Illumina (San Diego, CA) and GeneSeek (Lincoln, NE); and (5) SNP showing Mendelian inconsistencies within 44 trios (dam, sire, and offspring) and the International Mapping Flock [51]. A total of 5,207 were removed (Table S3), leaving 49,034 SNP. Genotypes are available formatted for analysis in PLINK [52] from the ISGC website [53].

### Genetic Diversity

Five metrics were used to estimate levels of within-breed genetic diversity (Table S4). The proportion of polymorphic SNP (*P*_n_) gives the fraction of total SNP that displayed both alleles within each population. Expected heterozygosity (*H*_e_) and the inbreeding coefficient (*F*) were estimated using PLINK [52], while allelic richness (*A*_r_) and private allele richness (p*A*_r_) were estimated by ADZE [54].

### Analysis of Ascertainment Bias

Analysis of allele frequency distributions, plotted separately for SNP identified by Roche 454 and Illumina GA sequencing, indicated the presence of ascertainment bias (Figure S1). To determine its effect on estimates of genetic relatedness between populations, Reynold's distance was calculated between breeds using five different subsets of SNP (Figure S11). The SNP sets were as (i) all 49,034 SNP, (ii) 33,115 SNP identified using Roche 454, (iii) 15,427 SNP identified using Illumina GA, and (iv) 22,678 SNP identified by application of LD pruning using PLINK–indep (50 5 0.05). This calculated LD between SNP in windows containing 50 markers before removing one SNP from each pair where LD exceeded 0.05 and (v) 20,279 SNP polymorphic in non-domestic sheep that were SNP pruned using LD as described for (iv). The resulting five NeighborNet trees were almost identical, indicating ascertainment bias did not have a large impact on the interpretations based on genetic distance. The removal of SNP in high LD has been shown to counter the effect of ascertainment bias and generate meaningful comparisons between populations [10]. LD-based pruning as described above preferentially reduced mean SNP heterozygosity within European populations used heavily during SNP discovery.

### Analysis of Genetic Structure

In order to understand the relationship within and between breeds across each major geographic group, Principal Components Analysis (PCA) was performed using EigenStrat [55]. Initial PCA using all 2,819 animals revealed six breeds containing in excess of 100 animals skewed the clustering. This prompted a reduction in the number of animals used, where 1,612 animals were randomly selected to ensure 26 or fewer animals were included per breed (Figures 2 and S3). To ensure uncorrected LD did not distort the PCA [55], SNP pruning was used to identify two SNP sets. First, all 49,034 markers were subjected to LD-based pruning (>0.05) using PLINK to identify 22,678 SNP. Secondly, 32,847 SNP that retain polymorphism within wild feral sheep were subjected to the same LD-based SNP pruning (>0.05) to identify 20,279 SNP. The PCA results obtained did not differ significantly dependent on the SNP set used. Model-based clustering was performed using the admixture model, correlated allele frequencies, and 15,000 burnin and 35,000 simulation cycles in STRUCTURE version 2.3 [18]. Convergence was checked using two runs for each value of *K* (number of subpopulations). For supervised clustering, prior population information was introduced from six meta-populations consisting of regional pool of breeds considered to represent ancestral populations. The same meta-populations were used for updating the allele frequencies during the simulations. NeighborNet graphs were constructed from a matrix of Reynolds' distances using Splitstree [56].

### Estimates of Historic Effective Population Size, Extent of LD, Haplotype Sharing, and Divergence Times Between Breeds

To estimate historic effective population size for each breed, the degree of linkage disequilibrium (LD) was calculated as *r^2^* between all SNP pairs where MAF for each SNP in the pair was >0.10. *r^2^* values were grouped into bins based on the distance between SNP from the physical map. *N_t_* was then calculated as (1−*r^2^*)/(4*cr^2^*), where *c* is the distance between the SNPs in Morgans (we assumed 100 Mb = 1 Morgan) and *N_t_* is the effective population size *t* generations ago, where *t* = 1/2*c*. The most recent estimate of effective size was taken as *N_t_* when c = 1 Mb. We performed simulations to assess the sensitivity of the estimates of effective population size over generations based on LD, in populations with and without admixture events (Figure S6). A mutation-drift model was used in the simulations following [22]. The population consisted of individuals made up of a chromosome segment 50 Mb long with 6,901 SNP. A population of individuals was simulated with an initially very large population size 10,000 generations ago, declining to a small effective population size in recent generations. In the final 420 generations, the population was split into two “breeds.” In the non-admixed population, there was complete divergence between the breeds for the 420 generations. In the first admixed population, there was an admixture event, with crossing between the breeds (matings chosen at random across the two breeds) 220 generations ago. The admixing lasted 20 generations, after which the breeds diverged for a further 200 generations, with no more admixture events. LD (r^2^) was calculated between all marker papers and *N*e estimated at different times in the past as described for the real data. Five replicate simulations were performed for each scenario. The extent of haplotype sharing among populations was characterised with the *r* statistic, where *r* is a signed *r^2^* [19]. A high correlation between *r* values for all locus pairs separated by the same physical distance among two breeds requires that the same haplotypes are found within both breeds. This means the sign of the *r* statistic is preserved across breeds only if the phase relationship among alleles is the same in both populations (leading to a high value for *r* if this is the case). The correlation of *r* between breeds was calculated for SNP separated by <10 kb, 10–25 kb, 25–50 kb, 50–100 kb, and 100–250 kb (Figures S7, S8, S9). There will be some error in calculating the correlation of *r* between two breeds due to finite sampling of haplotypes within a breed (e.g., limited sample size). To determine the extent of this error, we calculated the correlation of the *r* values at these different lengths of haplotypes for the Merino and Industry Merino samples, which are samples from the same breed. This gave a correlation between the *r* values for each bin size of 0.6. All correlations of *r* values for all breed comparisons were then divided by 0.6 to correct for sampling. Only corrected values are presented. As detailed in [19] the change in correlation of *r* between two breeds with increasing marker distance can be used to estimate generations since divergence from a common ancestral population. From [19], the expectation for *r* after *T* generations of divergence is *E*(*r*_T_) = *e* ^−2*cT*^. The natural logarithm of the expected correlation of *r* then follows a linear decrease as a function of distance with slope −2*T*, and this was used to calculate divergence time between all breeds (Figure S10).

### Detection of Genomic Regions Under Selection

Global *F*_ST_ was calculated as described by [57]. Raw values were ranked and used to identify regions under position selection. Centred on the top SNP (0.1%), neighbouring markers were included until consecutive markers were encountered ranking outside of the top 5%. The second marker was excluded and the Mb position of each region was determined using sheep genome assembly version 1. SNP-specific *F*_ST_ values were smoothed using a local variable bandwidth estimator as described in [35] and plotted as a line in Figures 6 and 7. To identify genomic regions with shared selection signals across breeds, raw *F*_ST_ within each population was smoothed into 500 genomic divisions (98 SNP per region). The number of breeds with smoothed *F*_ST_ in excess of one standard deviation of the mean was plotted for values at each tail of the distribution. Analysis was performed to identify gene ontology (GO) terms that were significantly overrepresented in 181 genes residing within the 31 regions under selection (Table 1). The terms associated with the 181 genes were compared against a background set of 11,098 genes. Each of the 11,098 genes contain a SNP present on the SNP50 Beadchip, or a SNP within 2.5 Kb. Comparison of the two gene lists (target and background) was performed using the software GOrilla, which implements a hypergeometric distribution and mHG *p* value approach to determine significance [58].

## Supporting Information

Figure S1 Minor allele frequency (MAF) based on SNP type. MAF was estimated for each of five geographically defined breed groupings separately using either 33,115 SNP derived using Roche 454 (top panel) or 15,427 SNP derived using Illumina GAII (bottom panel). The breed membership of each group is given in Table S4 and the percentage of SNP is plotted for each frequency bin. The excess of low MAF SNP (<0.1) present within African and Asia breeds was more pronounced within the 454 derived SNP when compared to those obtained using Illumina GA sequencing. This does not reflect differences between sequencing technologies, but rather the composition of animals used during the two SNP discovery experiments. The 454 SNP were discovered using six animals, none of which were sampled from Africa or Asia. The GA SNP were discovered using 60 animals, 21 of which were drawn from Africa and Asia (Table S2).
(TIF)

Figure S2 Diversity between breeds compared using different SNP panels. Expected heterozygosity was calculated using four SNP panels: “49034” contains all SNP passing quality control (Table S3); “20279” was derived by pruning 32,847 SNP that retain polymorphism within wild feral sheep using LD (refer to the Materials and Methods section for detail); “454” contains 33,115 SNP ascertained using a small discovery panel; and “GAII” contains 15,247 SNP ascertained using a larger and genetically diverse discovery panel. Bold lines indicate breeds used in SNP discovery, and colours are used to indicate breed origin. Comparison of heterozygosity obtained from each panel revealed common SNP enriched within the 20279 panel produced the highest values for each breed, and that 454 SNP returned higher values than the GAII SNP in most breeds. Importantly, while the absolute value of heterozygosity was dependent on the SNP panel used, the ranking of breeds remained generally stable when calculated using different panels. This indicates conclusions concerning relative diversity between breeds and regions are unlikely to be heavily influenced by ascertainment bias.
(TIF)

Figure S3 Principal components analysis (PCA) of European-derived sheep. To visualise the complex relationships between European-derived populations, PCA was performed separately using northern European breeds (880 animals, left panel) and central and southern European breeds (438 animals, right panel). The inbred Soay, Boreray, and Macarthur Merino have been omitted. In the left panel, PC1 and PC2 resolve all British and most central European breeds and show the intermediate position of the Swiss crossbred Swiss Alpine, Swiss Mirror, and Swiss Brown-Black Mountain sheep. Individuals from three geographically distinct populations of Texel formed a single cluster (German, New Zealand, and Scottish Texel, Table S1). This indicates that even where sampling is very broad, individuals from the same breed form a single cluster separate from other related breeds. In the right panel most Central European, Merino, and Mediterranean breeds were resolved. PC3 separates Rambouillet, Chinese Merino, and Australian Merino. PC1 to PC4 did not resolve the Australian, Australian Polled, and the Australian Industry Merino and only partially the Italian and Spanish breeds.
(TIF)

Figure S4 Model-based clustering of sheep. The diverse origin of the sheep genome was examined by model-based clustering using STRUCTURE [18]. This visualized a decomposition of the genome in a predefined number of *K* components, which may correspond to the genomes of ancestral populations. Representative results are shown for unsupervised clustering using *K* = 2, 5, and 8. At *K* = 2, a marked division is observed between European and Asian breeds, which corresponded to the first principal component in PCA (Figure 2). For increasing values of *K* up to *K* = 9, the results were reproducible and revealed clustering into British, Mediterranean (Southern and Western European), South-West Asian (Middle Eastern), Asian, and African breeds. Separate clusters were assigned to the Soay and Boreray (at *K* = 5), Dorset (at *K* = 6), Friesian (*K* = 7), and Wiltshire breeds (*K* = 9). The admixed nature of Brazilian and Caribbean animals can be seen at higher values of *K*. Essentially the same patterns were obtained with the Admixture program [59] using either 20,279 SNP or 22,678 SNP datasets, although some clusters appeared at different *K* (unpublished data). Supervised clustering (S, bottom panel) was performed using six predefined regional genomic components reconstructed by pooling breeds as indicated by the coloured horizontal line.
(TIF)

Figure S5 Proportion of variance explained in principal component analysis. Genetic distance between each pair of animals in the global sheep diversity panel was analysed using PCA. The proportion of variance explained by each of the first 50 PCs is given at left (blue diamonds) and the cumulative proportion is shown at right (red triangles). The first PC explained approximately 3% of the variance, while the first 20 PCs together explain 16.3% of the total variation.
(TIF)

Figure S6 Effective population size inferred from linkage disequilibrium (r2) with and without an admixture event in a simulated sheep population. The decline in effective population size was simulated to be similar to that observed in the real HapMap data for many breeds. The admixture event occurred 220 to 200 generations ago and lasted 20 generations before the breeds diverged for a further 200 generations without further admixture. LD (r2) was calculated between SNP and *N*e was estimated at different times in the past as described for the real data. This shows the admixture event did affect the inferred *N*e, with a higher estimate for the generations in which the admixture event is occurring. However, impact on estimates of *N*e for generations that were not within 100 generations of the admixture event were minimal. Further, the pattern of inferred *N*e for the admixed population suggests that there is information in the pattern of LD to pick up such events.
(TIF)

Figure S7 Extensive sharing of short-length haplotypes between breeds. The persistence of haplotype phase between breeds was evaluated using the signed *r* statistic [19]. The correlation of *r* was calculated using SNP pairs separately by short physical distances (0–10 Kb). The values are given as heat map, where each cell represents haplotype sharing between a breed pair. This revealed a high degree of conservation in LD phase between sheep populations.
(TIF)

Figure S8 Haplotypes sharing between breeds at intermediate physical distances. The persistence of haplotype phase between breeds was evaluated using the signed *r* statistic [19]. The correlation of *r* was calculated using SNP pairs separately by short physical distances (10–25 Kb). The values are given as heat map, where each cell represents haplotype sharing between a breed pair. The values were used to plot haplotype sharing in Figure 1C.
(TIF)

Figure S9 Long-distance haplotype sharing between breeds. The persistence of haplotype phase between breeds was calculated in the same way as in Figure S6 using SNP pairs separated by 100–250 kb. This revealed much lower levels of conservation in LD phase between breeds. Some pair-wise population comparisons retained high LD phase, including the three geographically dispersed populations of the Texel, the Merino and its derivatives, and finally selections lines within the same breed such as the Meat Lacaune and Milk Lacaune. Long-range haplotype sharing was also detected between Swiss breeds (e.g., SBS, SMS, and SWA) and both British and Merino-type breeds, suggesting a mixed origin.
(TIF)

Figure S10 Divergence time between breeds. Divergence time (in generations) between breeds was calculated from the extent of haplotype sharing that persists at increasing physical distance between SNP pairs [19]. Breed pairs separated by short divergence times are represented by dark squares, while breeds separated by longer divergence are given as progressively lighter squares. The divergence time values were used to generate the NeighborNet graph in Figure 3.
(TIF)

Figure S11 The effect of SNP ascertainment on breed relationships. To evaluate the effect of SNP ascertainment on visualised relationships between sheep breeds, NeighborNet graphs were constructed and compared using five different SNP sets. Graphs were constructed using all 49,034 SNP (A); 33,115 SNP identified using Roche 454 (B); 15,427 SNP identified using Illumina GA (C); 22,678 SNP identified by application of LD pruning of the full SNP set (D); and 20,279 SNP polymorphic in non-domestic sheep that displayed LD<0.05 (E). The topology of each graph is very similar, indicating SNP ascertainment doe not have a strong impact on interpretation of genetic relationships.
(TIF)

Table S1 Global Sheep Diversity Panel.
(DOC)

Table S2 SNP discovery for the *ovine* SNP50 BeadChip.
(DOC)

Table S3 Quality control filters used to remove SNP.
(DOC)

Table S4 Genetic diversity and recent effective population size.
(DOC)

Table S5 Enrichment analysis of gene ontology (GO) terms for genes in regions under selection.
(DOC)

Table S6 Selection signals identified in both sheep and cattle.
(DOC)
