# Supplementary figures and images for: Genome-Wide Analysis of the World's Sheep Breeds Reveals High Levels of Historic Mixture and Strong Recent Selection
Source: PLoS Biol. 2012 Feb 7;10(2):e1001258. doi: 10.1371/journal.pbio.1001258 (PMC3274507; doi:10.1371/journal.pbio.1001258)

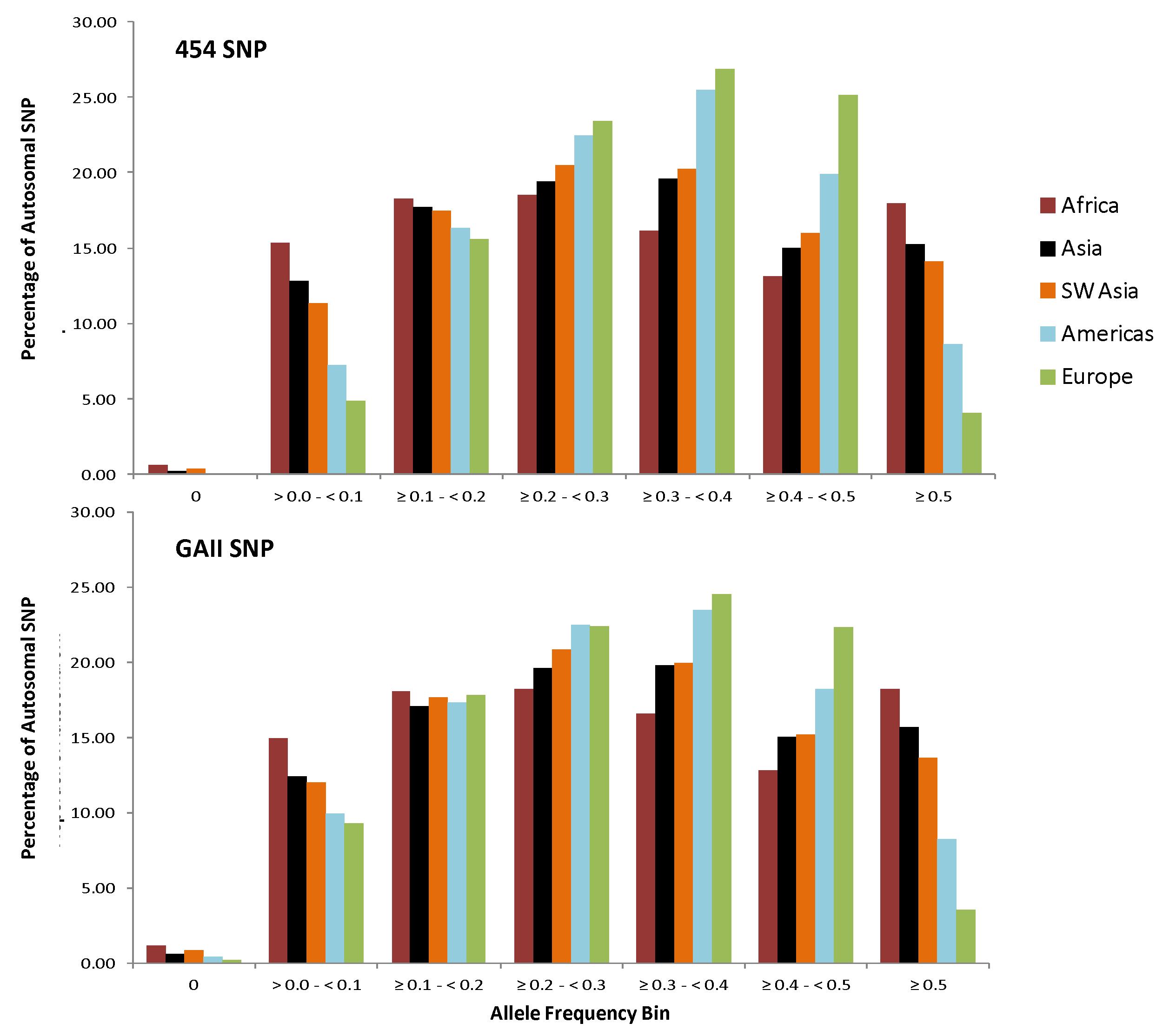

Supplement: Figure S1 — Minor allele frequency (MAF) based on SNP type. MAF was estimated for each of five geographically defined breed groupings separately using either 33,115 SNP derived using Roche 454 (top panel) or 15,427 SNP derived using Illumina GAII (bottom panel). The breed membership of each group is given in Table S4 and the percentage of SNP is plotted for each frequency bin. The excess of low MAF SNP (<0.1) present within African and Asia breeds was more pronounced within the 454 derived SNP when compared to those obtained using Illumina GA sequencing. This does not reflect differences between sequencing technologies, but rather the composition of animals used during the two SNP discovery experiments. The 454 SNP were discovered using six animals, none of which were sampled from Africa or Asia. The GA SNP were discovered using 60 animals, 21 of which were drawn from Africa and Asia (Table S2). (TIF) [file pbio.1001258.s001.tif]

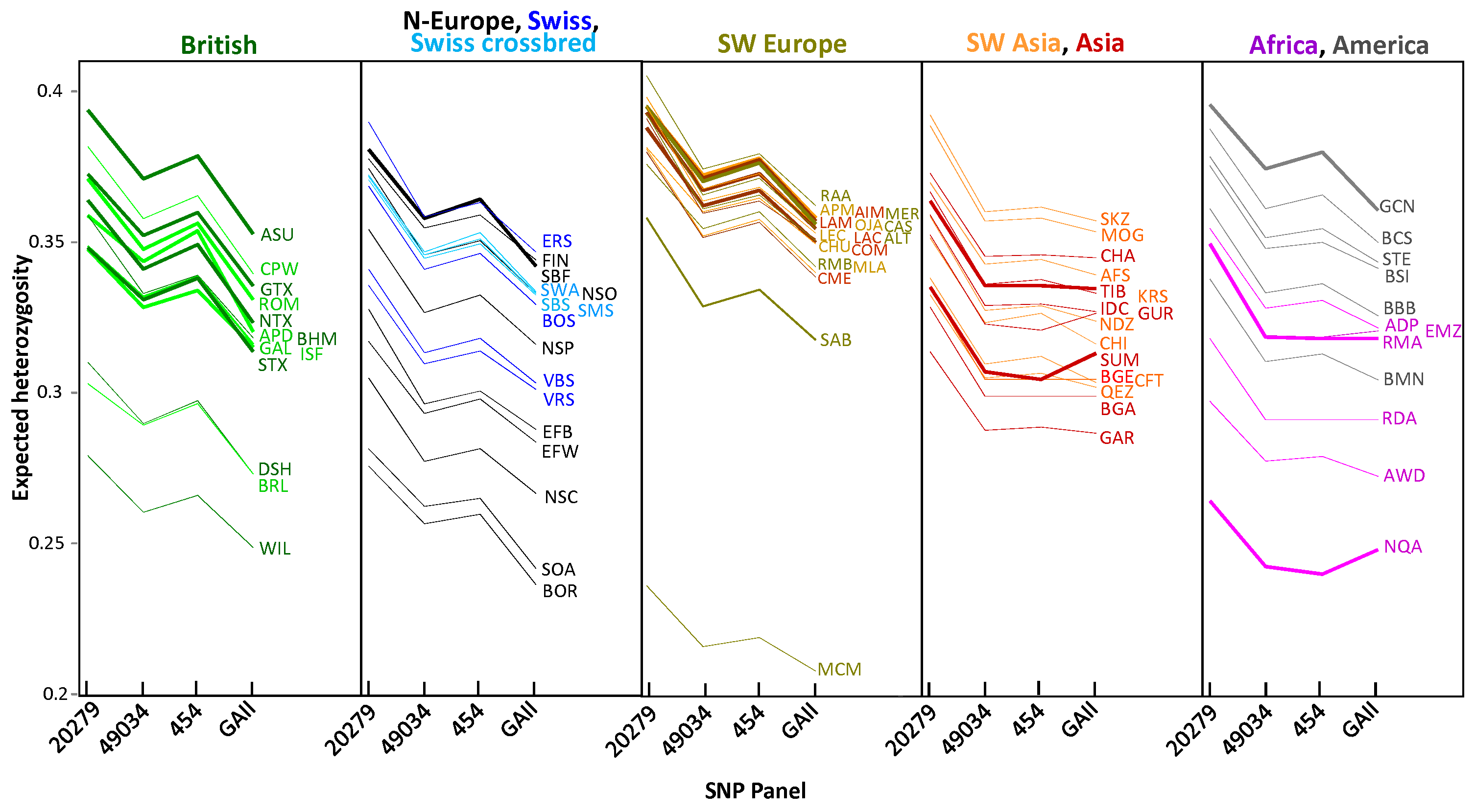

Supplement: Figure S2 — Diversity between breeds compared using different SNP panels. Expected heterozygosity was calculated using four SNP panels: “49034” contains all SNP passing quality control (Table S3); “20279” was derived by pruning 32,847 SNP that retain polymorphism within wild feral sheep using LD (refer to the Materials and Methods section for detail); “454” contains 33,115 SNP ascertained using a small discovery panel; and “GAII” contains 15,247 SNP ascertained using a larger and genetically diverse discovery panel. Bold lines indicate breeds used in SNP discovery, and colours are used to indicate breed origin. Comparison of heterozygosity obtained from each panel revealed common SNP enriched within the 20279 panel produced the highest values for each breed, and that 454 SNP returned higher values than the GAII SNP in most breeds. Importantly, while the absolute value of heterozygosity was dependent on the SNP panel used, the ranking of breeds remained generally stable when calculated using different panels. This indicates conclusions concerning relative diversity between breeds and regions are unlikely to be heavily influenced by ascertainment bias. (TIF) [file pbio.1001258.s002.tif]

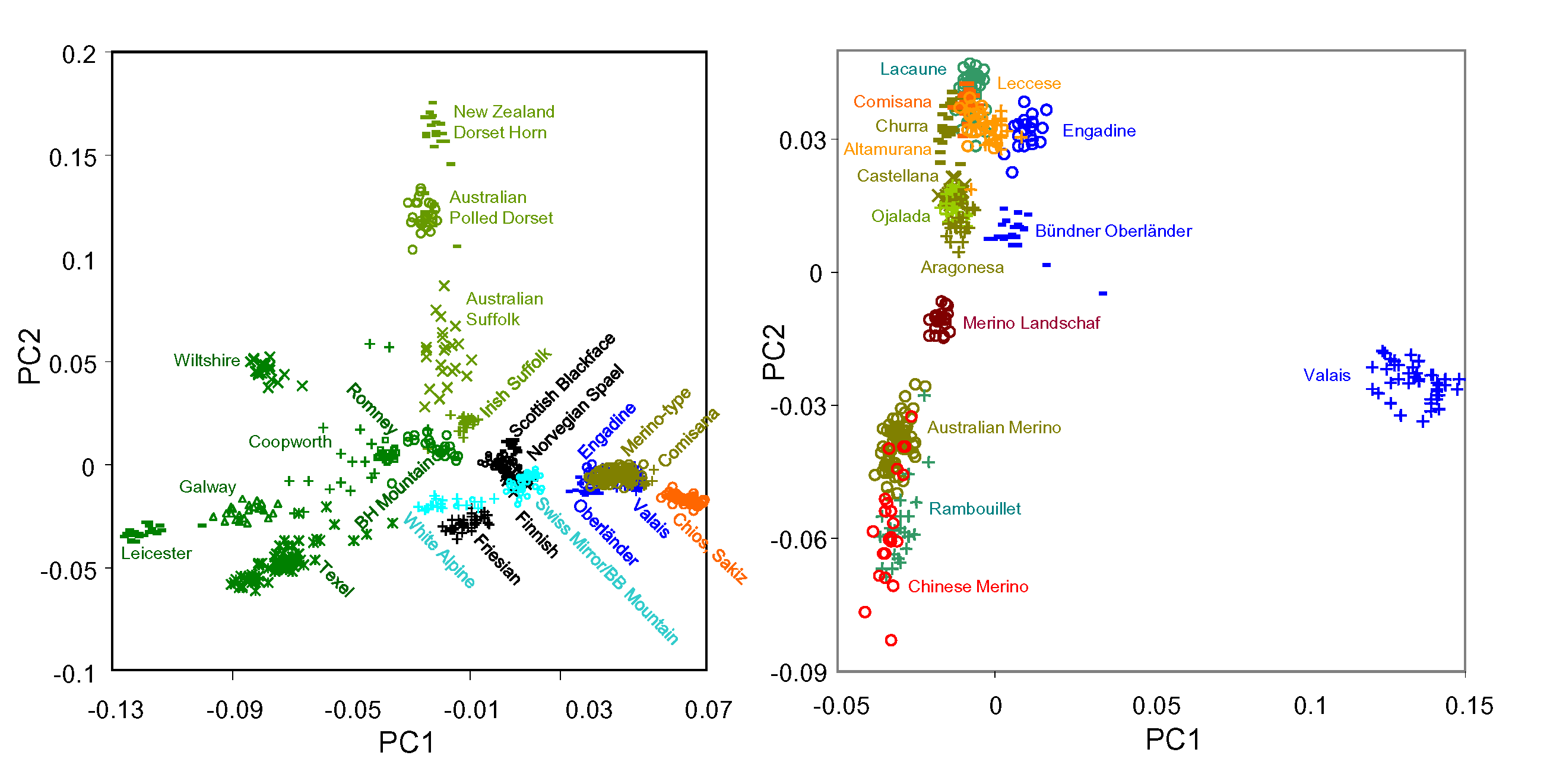

Supplement: Figure S3 — Principal components analysis (PCA) of European-derived sheep. To visualise the complex relationships between European-derived populations, PCA was performed separately using northern European breeds (880 animals, left panel) and central and southern European breeds (438 animals, right panel). The inbred Soay, Boreray, and Macarthur Merino have been omitted. In the left panel, PC1 and PC2 resolve all British and most central European breeds and show the intermediate position of the Swiss crossbred Swiss Alpine, Swiss Mirror, and Swiss Brown-Black Mountain sheep. Individuals from three geographically distinct populations of Texel formed a single cluster (German, New Zealand, and Scottish Texel, Table S1). This indicates that even where sampling is very broad, individuals from the same breed form a single cluster separate from other related breeds. In the right panel most Central European, Merino, and Mediterranean breeds were resolved. PC3 separates Rambouillet, Chinese Merino, and Australian Merino. PC1 to PC4 did not resolve the Australian, Australian Polled, and the Australian Industry Merino and only partially the Italian and Spanish breeds. (TIF) [file pbio.1001258.s003.tif]

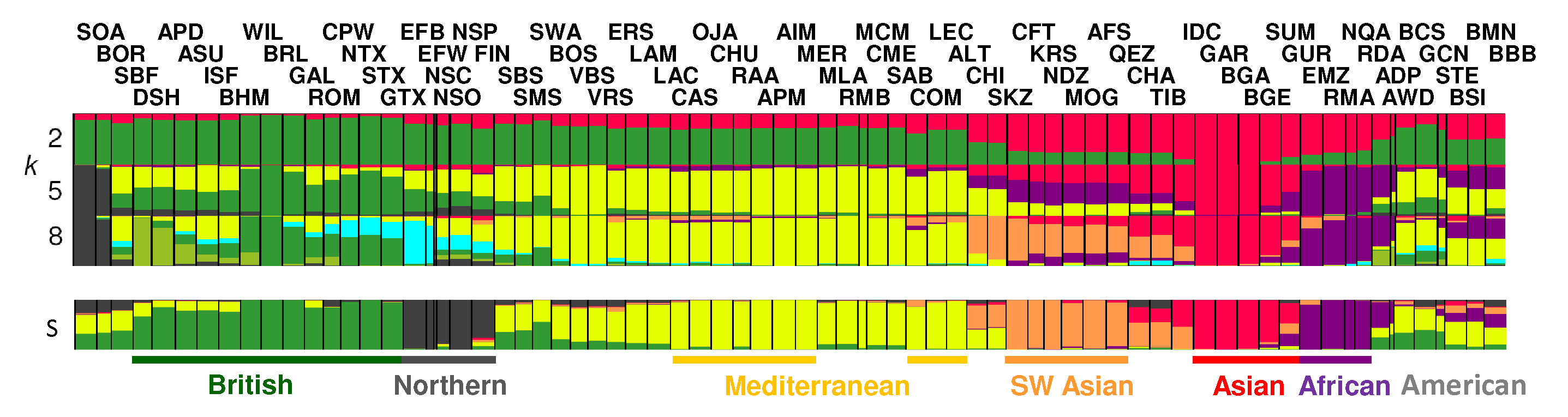

Supplement: Figure S4 — Model-based clustering of sheep. The diverse origin of the sheep genome was examined by model-based clustering using STRUCTURE [18]. This visualized a decomposition of the genome in a predefined number of K components, which may correspond to the genomes of ancestral populations. Representative results are shown for unsupervised clustering using K = 2, 5, and 8. At K = 2, a marked division is observed between European and Asian breeds, which corresponded to the first principal component in PCA (Figure 2). For increasing values of K up to K = 9, the results were reproducible and revealed clustering into British, Mediterranean (Southern and Western European), South-West Asian (Middle Eastern), Asian, and African breeds. Separate clusters were assigned to the Soay and Boreray (at K = 5), Dorset (at K = 6), Friesian (K = 7), and Wiltshire breeds (K = 9). The admixed nature of Brazilian and Caribbean animals can be seen at higher values of K. Essentially the same patterns were obtained with the Admixture program [59] using either 20,279 SNP or 22,678 SNP datasets, although some clusters appeared at different K (unpublished data). Supervised clustering (S, bottom panel) was performed using six predefined regional genomic components reconstructed by pooling breeds as indicated by the coloured horizontal line. (TIF) [file pbio.1001258.s004.tif]

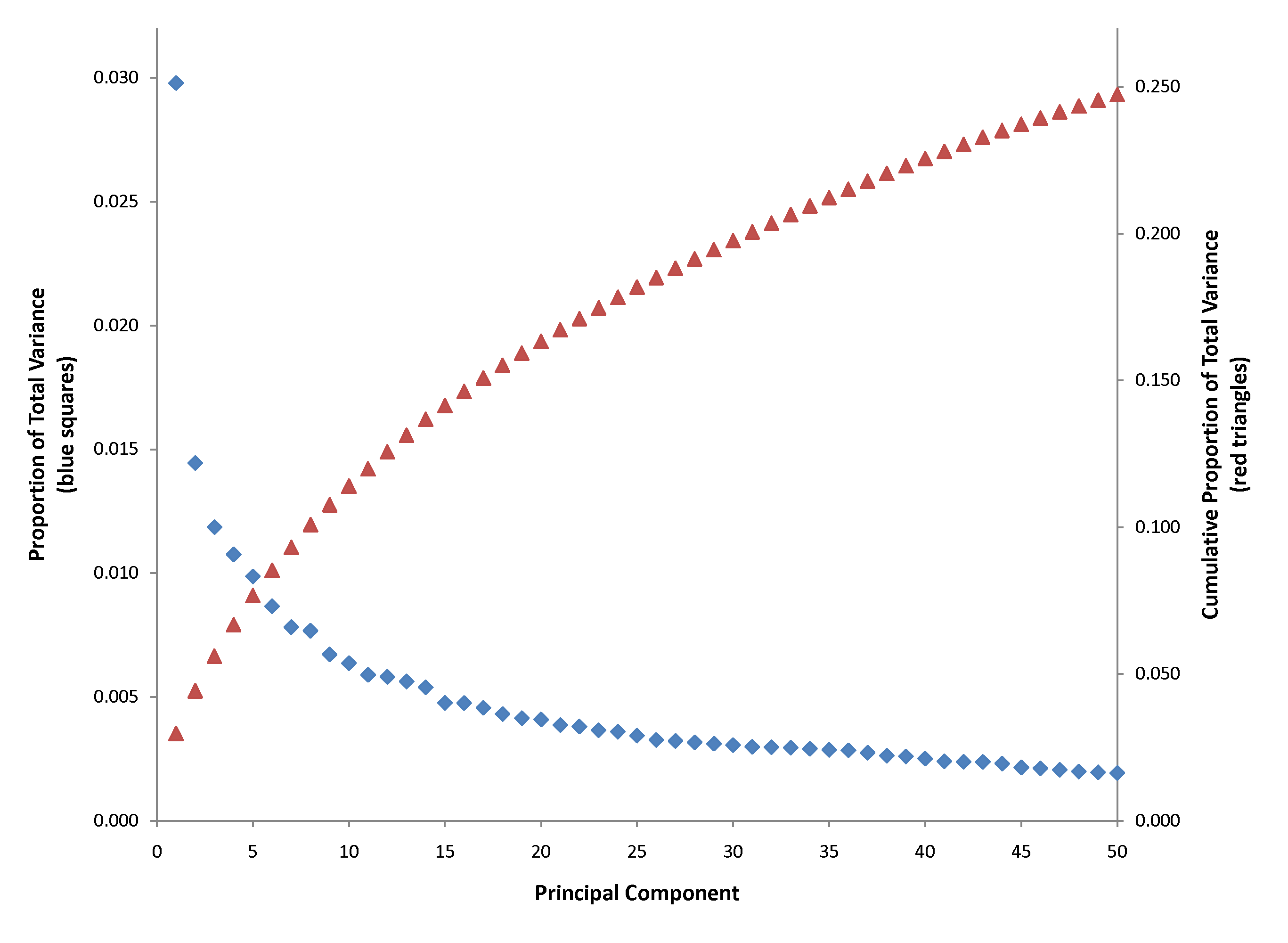

Supplement: Figure S5 — Proportion of variance explained in principal component analysis. Genetic distance between each pair of animals in the global sheep diversity panel was analysed using PCA. The proportion of variance explained by each of the first 50 PCs is given at left (blue diamonds) and the cumulative proportion is shown at right (red triangles). The first PC explained approximately 3% of the variance, while the first 20 PCs together explain 16.3% of the total variation. (TIF) [file pbio.1001258.s005.tif]

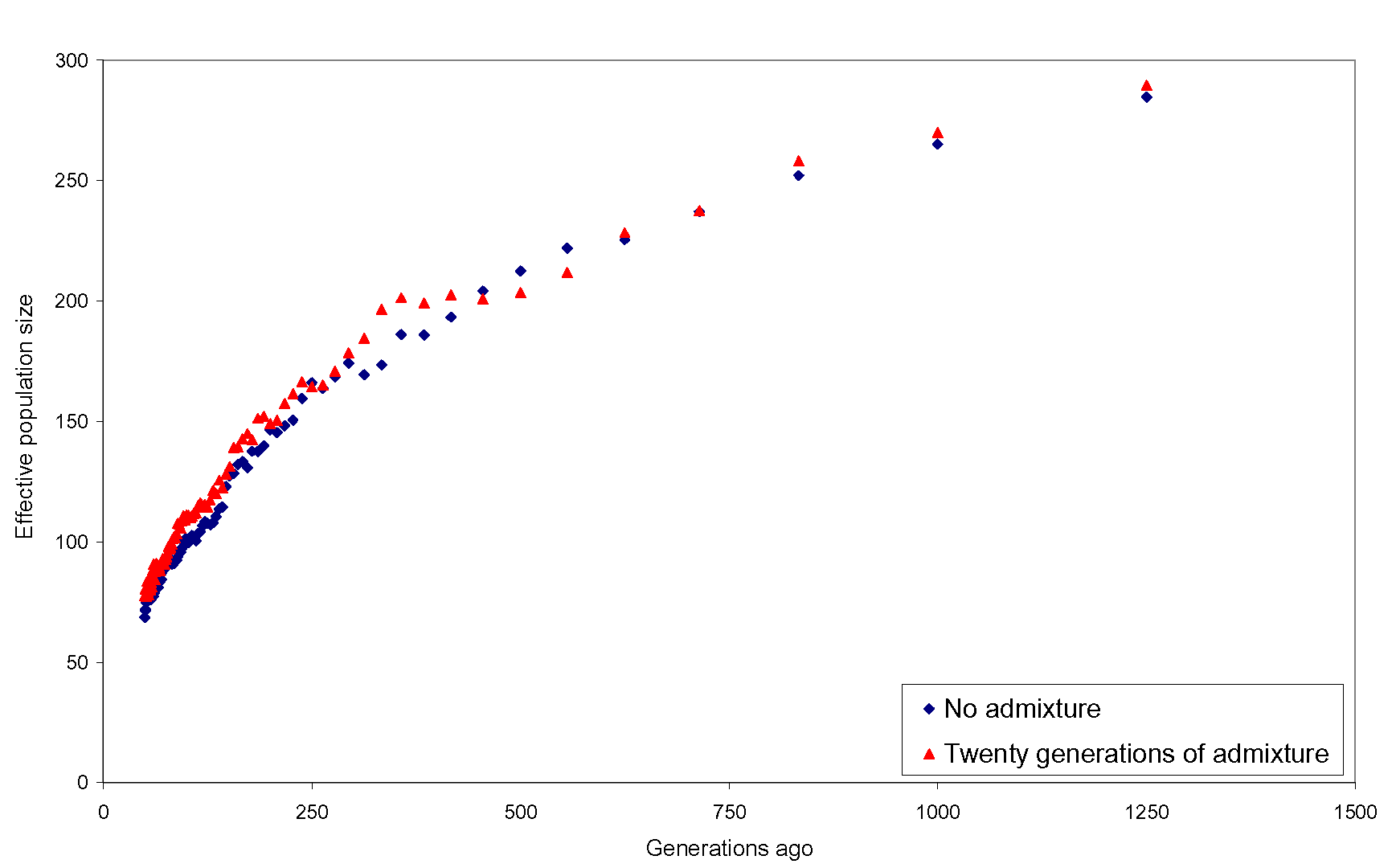

Supplement: Figure S6 — Effective population size inferred from linkage disequilibrium (r2) with and without an admixture event in a simulated sheep population. The decline in effective population size was simulated to be similar to that observed in the real HapMap data for many breeds. The admixture event occurred 220 to 200 generations ago and lasted 20 generations before the breeds diverged for a further 200 generations without further admixture. LD (r2) was calculated between SNP and Ne was estimated at different times in the past as described for the real data. This shows the admixture event did affect the inferred Ne, with a higher estimate for the generations in which the admixture event is occurring. However, impact on estimates of Ne for generations that were not within 100 generations of the admixture event were minimal. Further, the pattern of inferred Ne for the admixed population suggests that there is information in the pattern of LD to pick up such events. (TIF) [file pbio.1001258.s006.tif]

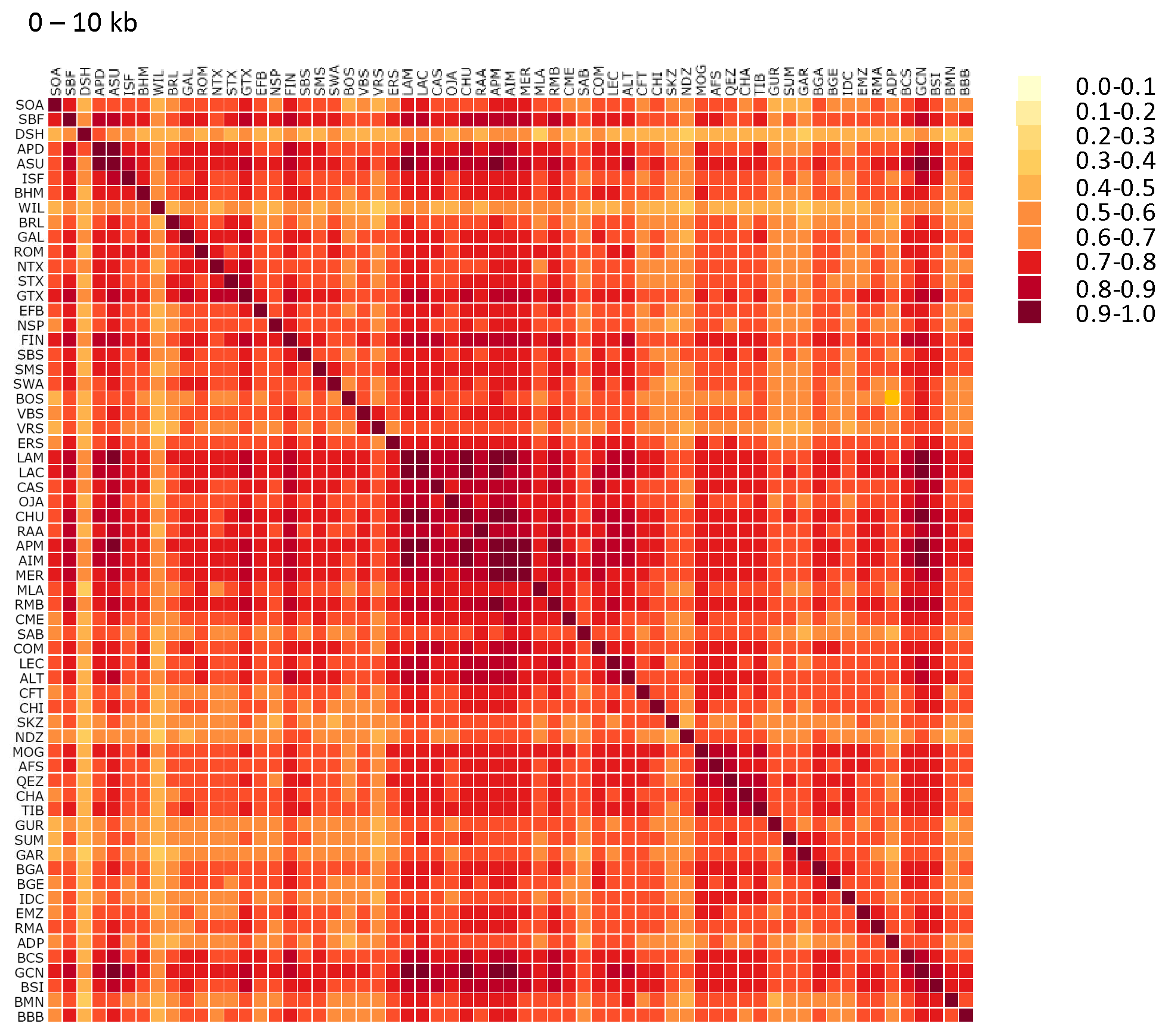

Supplement: Figure S7 — Extensive sharing of short-length haplotypes between breeds. The persistence of haplotype phase between breeds was evaluated using the signed r statistic [19]. The correlation of r was calculated using SNP pairs separately by short physical distances (0–10 Kb). The values are given as heat map, where each cell represents haplotype sharing between a breed pair. This revealed a high degree of conservation in LD phase between sheep populations. (TIF) [file pbio.1001258.s007.tif]

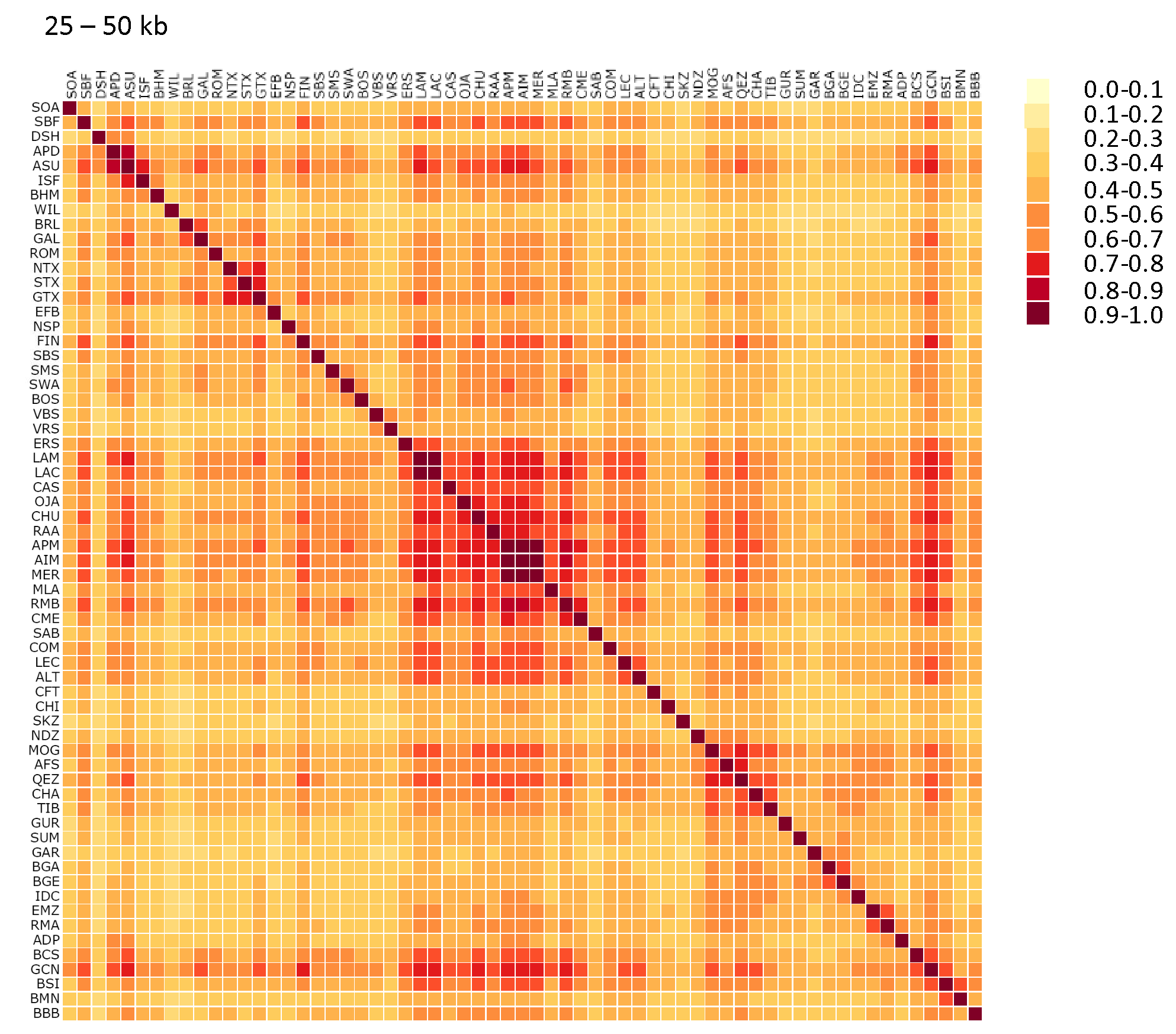

Supplement: Figure S8 — Haplotypes sharing between breeds at intermediate physical distances. The persistence of haplotype phase between breeds was evaluated using the signed r statistic [19]. The correlation of r was calculated using SNP pairs separately by short physical distances (10–25 Kb). The values are given as heat map, where each cell represents haplotype sharing between a breed pair. The values were used to plot haplotype sharing in Figure 1C. (TIF) [file pbio.1001258.s008.tif]

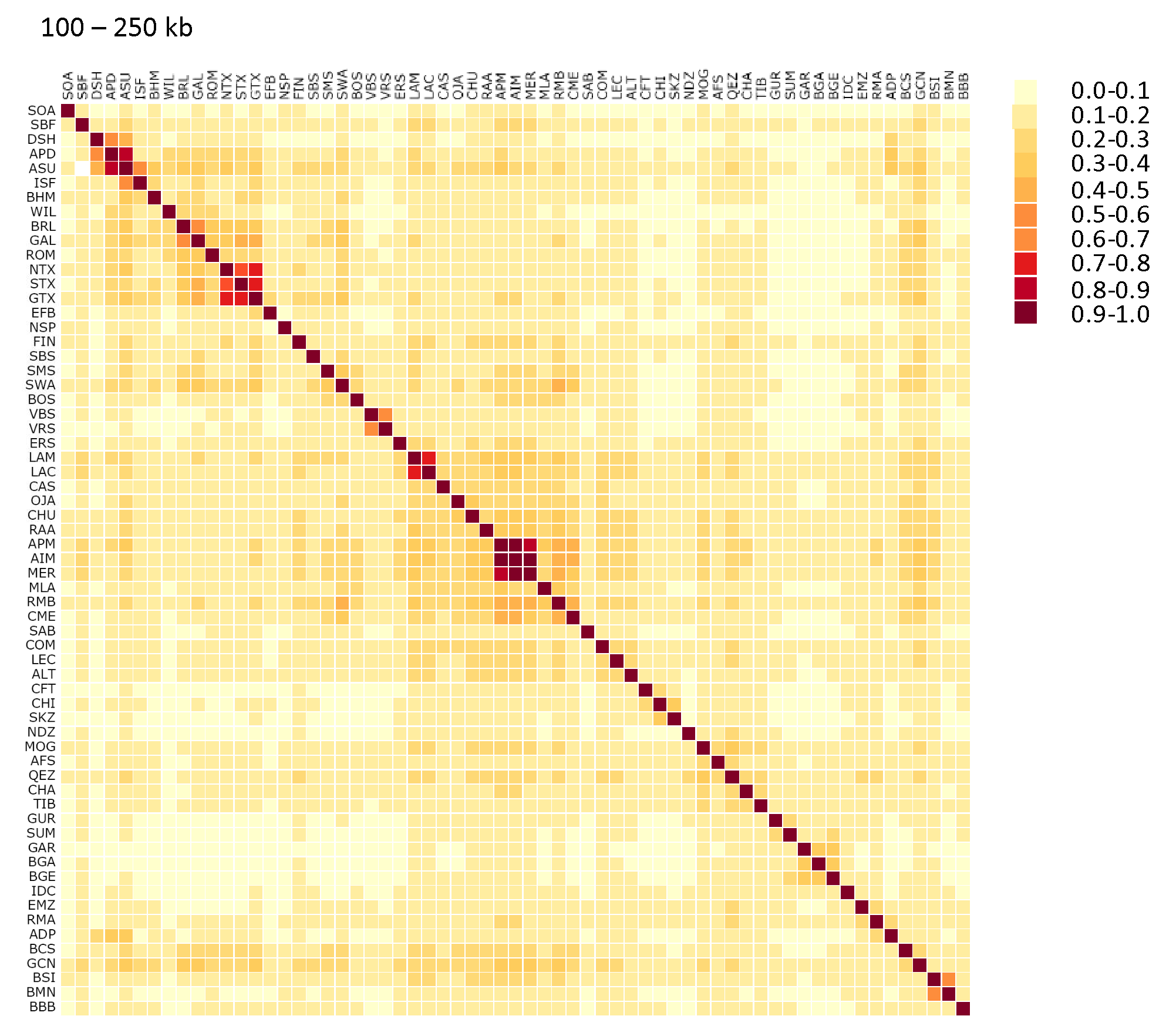

Supplement: Figure S9 — Long-distance haplotype sharing between breeds. The persistence of haplotype phase between breeds was calculated in the same way as in Figure S6 using SNP pairs separated by 100–250 kb. This revealed much lower levels of conservation in LD phase between breeds. Some pair-wise population comparisons retained high LD phase, including the three geographically dispersed populations of the Texel, the Merino and its derivatives, and finally selections lines within the same breed such as the Meat Lacaune and Milk Lacaune. Long-range haplotype sharing was also detected between Swiss breeds (e.g., SBS, SMS, and SWA) and both British and Merino-type breeds, suggesting a mixed origin. (TIF) [file pbio.1001258.s009.tif]

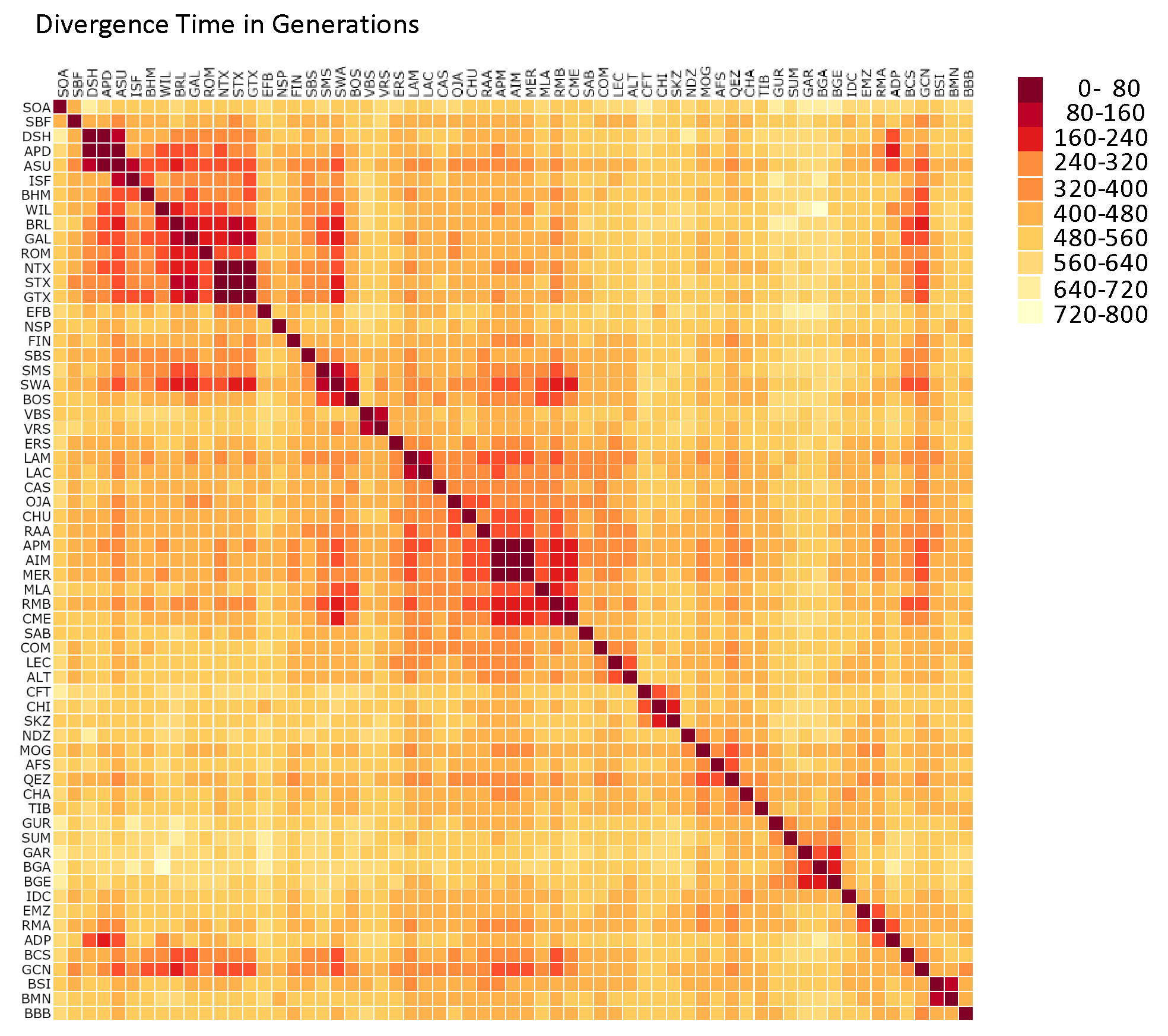

Supplement: Figure S10 — Divergence time between breeds. Divergence time (in generations) between breeds was calculated from the extent of haplotype sharing that persists at increasing physical distance between SNP pairs [19]. Breed pairs separated by short divergence times are represented by dark squares, while breeds separated by longer divergence are given as progressively lighter squares. The divergence time values were used to generate the NeighborNet graph in Figure 3. (TIF) [file pbio.1001258.s010.tif]

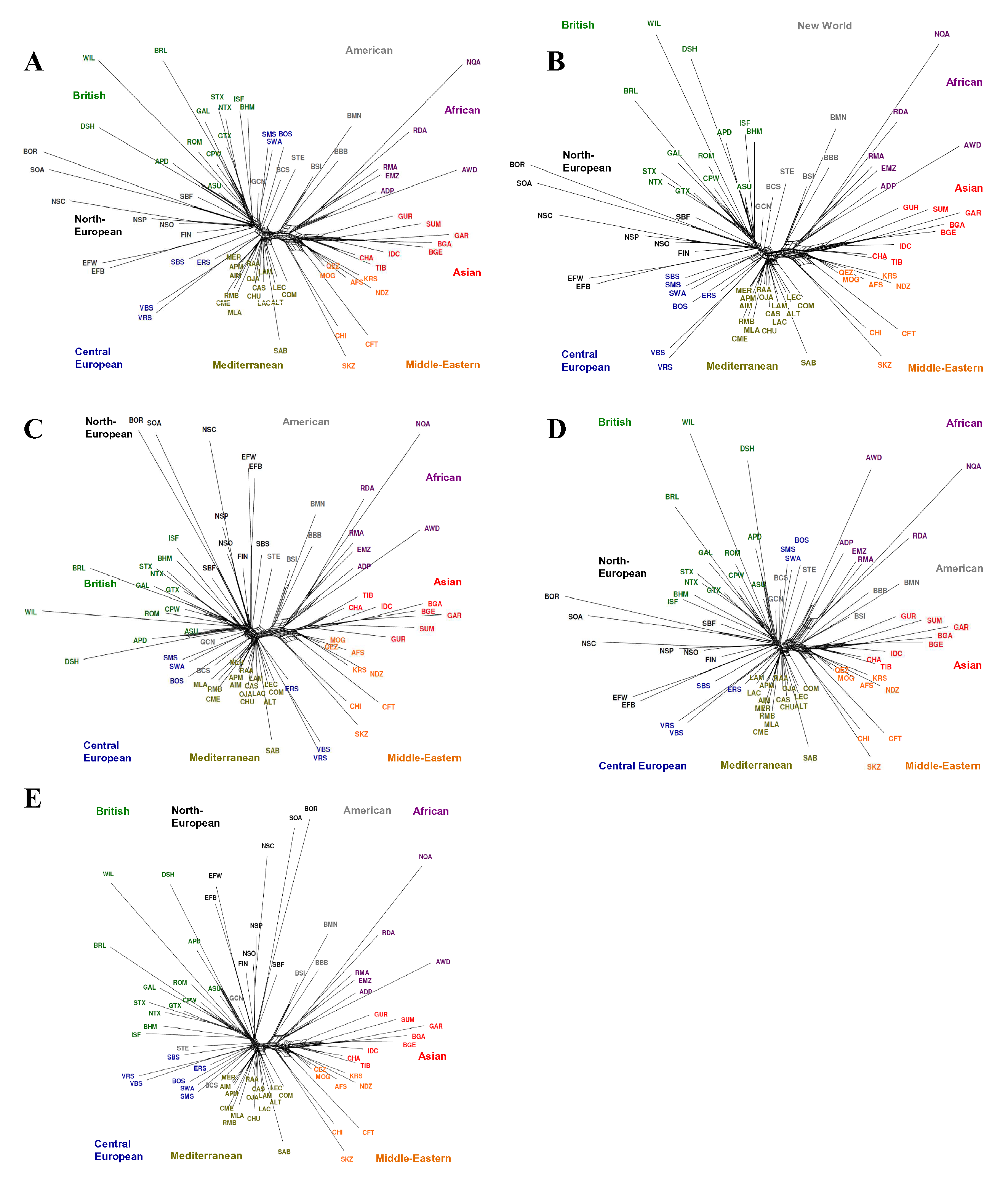

Supplement: Figure S11 — The effect of SNP ascertainment on breed relationships. To evaluate the effect of SNP ascertainment on visualised relationships between sheep breeds, NeighborNet graphs were constructed and compared using five different SNP sets. Graphs were constructed using all 49,034 SNP (A); 33,115 SNP identified using Roche 454 (B); 15,427 SNP identified using Illumina GA (C); 22,678 SNP identified by application of LD pruning of the full SNP set (D); and 20,279 SNP polymorphic in non-domestic sheep that displayed LD<0.05 (E). The topology of each graph is very similar, indicating SNP ascertainment doe not have a strong impact on interpretation of genetic relationships. (TIF) [file pbio.1001258.s011.tif]
