# Supplementary material for: Genome-Wide Analysis of the World's Sheep Breeds Reveals High Levels of Historic Mixture and Strong Recent Selection
Source: PLoS Biol. 2012 Feb 7;10(2):e1001258. doi: 10.1371/journal.pbio.1001258 (PMC3274507; doi:10.1371/journal.pbio.1001258)
Supplement: Table S1 — Global Sheep Diversity Panel. (DOC) [file pbio.1001258.s012.doc]

**Table S1.** Global Sheep Diversity Panel

|  |  |  |  |  |
| --- | --- | --- | --- | --- |
|  |  |  |  |  |
| **Breed** | **Abbrev** | **Number** | **Origin** | **Contributor** |
|  |  |  |  |  |
| African Dorper | ADP | 21 | Africa | Mikka Tapio |
| African White Dorper | AWD | 6 | Africa | James Kijas |
| Afshari | AFS | 37 | SW Asia | Henner Simianer |
| Altamurana | ALT | 24 | SW Europe | Elena Ciani |
| Australian Coopworth | CPW | 19 | Northern Europe | James Kijas |
| Australian Industry Merino | AIM/MER | 88 | SW Europe | James Kijas |
| Australian Merino | MER | 50 | SW Europe | James Kijas |
| Australian Poll Dorset | APD | 108 | Northern Europe | James Kijas |
| Australian Poll Merino | APM/MER | 98 | SW Europe | James Kijas |
| Australian Suffolk | ASU/SUF | 109 | Northern Europe | James Kijas |
| Bangladeshi BGE | BGE | 24 | Asia | Faruque Mdomar |
| Bangladeshi Garole | BGA | 24 | Asia | Faruque Mdomar |
| Barbados Black Belly | BBB | 24 | Americas | Cyril Roberts |
| Black-Headed Mutton | BHM | 24 | Central Europe | Ottmar Distl |
| Border Leicester | BRL | 48 | Northern Europe | James Kijas |
| Boreray | BOR | 17 | Northern Europe | Josephine Pemberton |
| Brazilian Creole | BCS | 23 | Americas | Samuel Paiva |
| Bundner Oberlander Sheep | BOS | 24 | Central Europe | Cord Drogemuller |
| Castellana | CAS | 23 | SW Europe | JJ Arranz |
| Changthangi | CHA | 29 | Asia | Jorn Benenwitz |
| Chinese Merino | CME | 23 | SW Europe | Runlin Ma |
| Chios | CHI | 23 | SW Europe | Georgios Banos |
| Churra | CHU | 120 | SW Europe | JJ Arranz |
| Comisana | COM | 24 | SW Europe | Fabio Pila |
| Cyprus Fat Tail | CFT | 30 | SW Asia | Despoina Miltiadou |
| Deccani | IDC | 24 | Asia | Vidya Gupta |
| Dorset Horn | DSH | 21 | Northern Europe | John McEwan |
| East-Friesian Brown | EFB | 39 | Central Europe | Ottmar Distl |
| East-Friesian White | EFW | 9 | Central Europe | Ottmar Distl |
| Engadine Red Sheep | ERS | 24 | Central Europe | Cord Drogemuller |
| Ethiopian Menz | EMZ | 34 | Africa | Mikka Tapio |
| Finn sheep | FIN | 99 | Northern Europe | Juha Kantanen |
| Galway | GAL | 49 | Northern Europe | David Machugh |
| Garut | GUR | 22 | Asia | Herman Raadsma |
| German Texel | GTX/TEX | 46 | Northern Europe | Cord Drogemuller |
| Gulf Coast Native | GCN | 94 | Americas | Noelle Cockett |
| Indian Garole | GAR | 26 | Asia | Vidya Gupta |
| Irish Suffolk | ISF/SUF | 55 | Northern Europe | David Machugh |
| Karakas | KRS | 18 | SW Asia | Ibrahim Cemal |
| Leccese | LEC | 24 | SW Europe | Elena Ciani |
| MacArthur Merino | MCM | 10 | SW Europe | James Kijas |
| Meat Lacaune | LAM/LAC | 78 | SW Europe | Carole Moreno |
| Merino Landschaf | MLA | 24 | SW Europe | Georg Erhardt |
| Milk Lacaune | LAC | 103 | SW Europe | Carole Moreno |
| Moghani | MOG | 34 | SW Asia | Henner Simianer |
| Morada Nova | BMN | 22 | Americas | Samuel Paiva |
| Namaqua Afrikaner | NQA | 12 | Africa | James Kijas |
| New Zealand Romney | ROM | 24 | Northern Europe | John McEwan |
| New Zealand Texel | NTX/TEX | 24 | Northern Europe | John McEwan |
| Norduz | NDZ | 20 | SW Asia | Ibrahim Cemal |
| Ojalada | OJA | 24 | SW Europe | JJ Arranz |
| Old Norwegian Spaelsau | NSO/NSP | 15 | Northern Europe | Matthew Kent |
| Qezel | QEZ | 35 | SW Asia | Henner Simianer |
| Rambouillet | RMB | 102 | SW Europe | Noelle Cockett |
| Rasa Aragonesa | RAA | 22 | SW Europe | Jorge Calvo |
| Red Maasai | RMA | 45 | Africa | Mikka Tapio |
| Ronderib Afrikaner | RDA | 17 | Africa | James Kijas |
| Sakiz | SKZ | 22 | SW Asia | Ibrahim Cemal |
| Santa Ines | BSI | 47 | Americas | Samuel Paiva |
| Sardinian Ancestral Black | SAB | 20 | SW Europe | Antonello Carta |
| Scottish Blackface | SBF | 56 | Northern Europe | Lutz Bunger |
| Scottish Texel | STX/TEX | 80 | Northern Europe | Lutz Bunger |
| Soay | SOA | 110 | Northern Europe | Josephine Pemberton |
| Spael-coloured | NSC/NSP | 3 | Northern Europe | Matthew Kent |
| Spael-white | NSP | 32 | Northern Europe | Matthew Kent |
| St. Elizabeth | STE | 10 | Americas | Cyril Roberts |
| Sumatra | SUM | 24 | Asia | Herman Raadsma |
| Swiss Black-Brow nMountain Sheep | SBS | 24 | Central Europe | Cord Drogemuller |
| Swiss Mirror Sheep | SMS | 24 | Central Europe | Cord Drogemuller |
| Swiss White Alpine Sheep | SWA | 24 | Central Europe | Cord Drogemuller |
| Tibetan | TIB | 37 | Asia | Han Jianlin |
| Valais Blacknose Sheep | VBS | 24 | Central Europe | Cord Drogemuller |
| Valais Red Sheep | VRS | 24 | Central Europe | Cord Drogemuller |
| Wiltshire | WIL | 23 | Northern Europe | John McEwan |
|  | Total | 2819 |  |  |
|  |  |  |  |  |

^1^ South-West Asia (SW Asia) refers to present day Turkey and Iran, also sometimes referred to as the Middle East. Breeds codes following a slash indicate breeds sampled on different locations and are used in Fig. 1A.
