# Supplementary material for: Genome-Wide Analysis of the World's Sheep Breeds Reveals High Levels of Historic Mixture and Strong Recent Selection
Source: PLoS Biol. 2012 Feb 7;10(2):e1001258. doi: 10.1371/journal.pbio.1001258 (PMC3274507; doi:10.1371/journal.pbio.1001258)
Supplement: Table S2 — SNP discovery for the ovine SNP50 BeadChip. (DOC) [file pbio.1001258.s013.doc]

**Table S2. SNP Discovery for the *ovine* SNP50 BeadChip**

| **SNP Type ^1^** | **Breed ^2^** | **N** | **Breed Development ^3^** |
| --- | --- | --- | --- |
| 454 | Poll Dorset | 1 | Northern Europe |
| 454 | Merino | 1 | SW Europe |
| 454 and Illumina GA | Awassi | 1 | SW Asia |
| 454 | Texel | 1 | Northern Europe |
| 454 | Romney | 1 | Northern Europe |
| 454 | Scottish Blackface | 1 | Northern Europe |
| Sanger | Poll Dorset | 1 | Northern Europe |
| Sanger | Merino | 1 | SW Europe |
| Sanger and Illumina GA | Awassi | 1 | SW Asia |
| Sanger | Lacaune | 1 | SW Europe |
| Sanger and Illumina GA | Red Masai | 1 | Africa |
| Sanger | Texel | 1 | Northern Europe |
| Sanger | Romney | 1 | Northern Europe |
| Sanger and Illumina GA | Katahdin | 1 | Americas |
| Sanger and Illumina GA | Gulf Coast Native | 1 | Americas |
| Illumina GA | American Suffolk | 5 | Northern Europe |
| Illumina GA | Scottish Blackface | 5 | Northern Europe |
| Illumina GA | Indonesian Thin Tail | 5 | Asia |
| Illumina GA | Italian Sarda | 5 | SW Europe |
| Illumina GA | Merino | 5 | SW Europe |
| Illumina GA | Poll Dorset | 5 | Northern Europe |
| Illumina GA | Romney | 5 | Northern Europe |
| Illumina GA | Sumatran Thin Tail | 5 | Asia |
| Illumina GA | Texel | 5 | Northern Europe |
| Illumina GA | Tibetan | 5 | Asia |
| Illumina GA | Namaqua Afrikaner | 5 | Africa |

^1^ SNP type is given as 454, Illumina GA or Sanger to reflect the sequencing technology used to identify the SNP sets arrayed on the *ovine* SNP50 BeadChip. The number of SNP of each type is as follows: 454 SNP (33,115 or 67.5 %); Illumina GA SNP (15,427 or 31.5 %) and Sanger SNP (492 or 1 %).

^2^ The breed of sheep used to develop each SNP type differed. Note 454 SNP were discovered primarily using animals of European origin, while the Illumina GA SNP were identified using a larger number of animals selected from multiple regions. The number of animals used per breed is given as N.

^3^ The geographic region where breed development occurred. This may be different from the sampling location for some breeds, for example the Romney which was sampled within New Zealand.
