# Supplementary material for: Genome-Wide Analysis of the World's Sheep Breeds Reveals High Levels of Historic Mixture and Strong Recent Selection
Source: PLoS Biol. 2012 Feb 7;10(2):e1001258. doi: 10.1371/journal.pbio.1001258 (PMC3274507; doi:10.1371/journal.pbio.1001258)
Supplement: Table S3 — Quality control filters used to remove SNP. (DOC) [file pbio.1001258.s014.doc]

**Table S3. Quality Control Filters Used to Remove SNP**

|  |  |  |  |  | |  | |  | |  | |  |
| --- | --- | --- | --- | --- | --- | --- | --- | --- | --- | --- | --- | --- |
|  |  |  |  | |  | |  | |  | |  | |
| **QC** | **Quality Control** | **SNP** | **SNP Overlap Between QC Filters** | | | | | | | |  | |
| **Filter** | **Criteria** | **Identified** | **1** | | **2** | | **3** | | **4** | | **5** | |
|  |  |  |  | |  | |  | |  | |  | |
|  |  |  |  | |  | |  | |  | |  | |
| 1 | < 0.99 Call Rate | 3612 |  | | 3244 (89%) | | 433 (12%) | | 62 (2%) | | 4 (<1%) | |
| 2 | Assay Abnormality | 4101 | 3244 (79%) | |  | | 433 (10%) | | 114 (3%) | | 4 (<1%) | |
| 3 | MAF < 0.01 | 1165 | 433 (37%) | | 433 (37%) | |  | | 0 | | 0 | |
| 4 | Discordant Genotypes | 125 | 62 (50%) | | 114 (91%) | | 0 | |  | | 0 | |
| 5 | Inheritance Problems | 11 | 4 (37%) | | 4 (37%) | | 0 | | 0 | |  | |
|  |  |  |  | |  | |  | |  | |  | |
| All | Any filter | 5207 |  | |  | |  | |  | |  | |
|  |  |  |  | |  | |  | |  | |  | |
|  |  |  |  |  | |  | |  | |  | |  |

SNP were pruned from the experiment using five quality control filters (refer to the materials and methods for a detailed description). The number of SNP identified using each filter is given, along with overlaps between filters. Many SNP were identified as problematic by multiple QC tests. For example, 89% of SNP which had less than 99% call rate (filter 1) also displayed some form of assay abnormality (filter 2). Similarly, almost all of the SNP (91 %) which returned discordant genotypes when tested separately by Illumina and GeneSeek (filter 4) had an assay abnormality (filter 2).
