# Supplementary material for: Genome-Wide Analysis of the World's Sheep Breeds Reveals High Levels of Historic Mixture and Strong Recent Selection
Source: PLoS Biol. 2012 Feb 7;10(2):e1001258. doi: 10.1371/journal.pbio.1001258 (PMC3274507; doi:10.1371/journal.pbio.1001258)
Supplement: Table S4 — Genetic diversity and recent effective population size. (DOC) [file pbio.1001258.s015.doc]

**Table S4. Genetic Diversity and Recent Effective Population Size**

|  |  |  |  |  |  |  |  |  | |  |
| --- | --- | --- | --- | --- | --- | --- | --- | --- | --- | --- |
|  |  |  |  |  |  |  |  |  |  | |
| **Breed** | **n** | ***P*_n_** | ***H*_e_** | **F** | ***A*_r_** | **p*A*_r_** | ***N*_e_** | ***HS*_MER_** |  | |
|  |  |  |  |  |  |  |  |  |  | |
| **Asia** |  |  |  |  |  |  |  |  |  | |
| Bangladeshi | 24 | 0.85 | 0.31 | 0.33 | 1.87 | 0.01 | 582 | 0.45 |  | |
| Bangladeshi Garole | 24 | 0.84 | 0.30 | 0.28 | 1.86 | 0.01 | 437 | 0.43 |  | |
| Changthangi | 29 | 0.93 | 0.35 | 0.17 | 1.96 | 0.02 | 684 | 0.57 |  | |
| Deccani | 24 | 0.91 | 0.33 | 0.16 | 1.93 | 0.02 | 837 | 0.49 |  | |
| Garut | 22 | 0.90 | 0.32 | 0.17 | 1.92 | 0.03 | 337 | 0.46 |  | |
| Indian Garole | 26 | 0.84 | 0.29 | 0.28 | 1.86 | 0.01 | 364 | 0.41 |  | |
| Sumatran | 24 | 0.89 | 0.31 | 0.24 | 1.91 | 0.04 | 336 | 0.45 |  | |
| Tibetan | 37 | 0.92 | 0.34 | 0.20 | 1.95 | 0.04 | 903 | 0.54 |  | |
|  |  |  |  |  |  |  |  |  |  | |
| **Africa** |  |  |  |  |  |  |  |  |  | |
| African Dorper | 21 | 0.91 | 0.33 | 0.14 | 1.93 | 0.00 | 264 | 0.48 |  | |
| African White Dorper | 6 | 0.73 | 0.28 | 0.17 | 1.75 | 0.01 | - | - |  | |
| Ethiopian Menz | 34 | 0.90 | 0.32 | 0.20 | 1.93 | 0.00 | 1260 | 0.50 |  | |
| Namaqua Afrikaner | 12 | 0.68 | 0.24 | 0.29 | 1.70 | 0.04 | - | - |  | |
| Red Maasai | 45 | 0.93 | 0.32 | 0.19 | 1.96 | 0.01 | 1070 | 0.54 |  | |
| Ronderib Afrikaner | 17 | 0.84 | 0.29 | 0.23 | 1.86 | 0.00 | - | - |  | |
|  |  |  |  |  |  |  |  |  |  | |
| **SW Asia** |  |  |  |  |  |  |  |  |  | |
| Afshari | 37 | 0.94 | 0.34 | 0.11 | 1.96 | 0.01 | 465 | 0.56 |  | |
| Cyprus Fat Tail | 30 | 0.88 | 0.31 | 0.18 | 1.90 | 0.03 | 203 | 0.47 |  | |
| Karakas | 18 | 0.91 | 0.34 | 0.12 | 1.93 | 0.02 | - |  |  | |
| Moghani | 34 | 0.95 | 0.36 | 0.10 | 1.97 | 0.00 | 825 | 0.62 |  | |
| Norduz | 20 | 0.90 | 0.33 | 0.12 | 1.92 | 0.00 | 285 | 0.44 |  | |
| Qezel | 35 | 0.95 | 0.36 | 0.11 | 1.98 | 0.01 | 1317 | 0.63 |  | |
| Sakiz | 22 | 0.84 | 0.31 | 0.17 | 1.86 | 0.00 | 165 | 0.43 |  | |
|  |  |  |  |  |  |  |  |  |  | |
| **The Americas** |  |  |  |  |  |  |  |  |  | |
| Barbados Black Belly | 24 | 0.91 | 0.33 | 0.20 | 1.94 | 0.01 | 374 | 0.53 |  | |
| Brazilian Creole | 22 | 0.94 | 0.36 | 0.18 | 1.97 | 0.01 | 371 | 0.63 |  | |
| Gulf Coast Native | 94 | 0.96 | 0.38 | 0.09 | 1.99 | 0.01 | 611 | 0.72 |  | |
| Morada Nova | 20 | 0.89 | 0.31 | 0.22 | 1.91 | 0.00 | 327 | 0.46 |  | |
| Santa Ines | 45 | 0.94 | 0.35 | 0.14 | 1.97 | 0.00 | 520 | 0.60 |  | |
| St Elizabeth | 10 | 0.91 | 0.35 | 0.06 | 1.93 | 0.00 | - | - |  | |
|  |  |  |  |  |  |  |  |  |  | |
| **Southern and Western Europe** |  |  |  |  |  |  |  |  |  | |
| Altamurana | 23 | 0.94 | 0.36 | 0.10 | 1.97 | 0.01 | 469 | 0.64 |  | |
| Australian Industry Merino | 88 | 0.96 | 0.37 | 0.10 | 1.99 | 0.00 | 853 | 1.00 |  | |
| Australian Poll Merino | 98 | 0.96 | 0.37 | 0.09 | 1.99 | 0.01 | 918 | 0.97 |  | |
| Australian Merino | 46 | 0.96 | 0.37 | 0.10 | 1.99 | 0.00 | 833 | 1.00 |  | |
| Castellana | 22 | 0.94 | 0.37 | 0.07 | 1.97 | 0.01 | 546 | 0.65 |  | |
| Chinese Merino | 23 | 0.94 | 0.35 | 0.08 | 1.96 | 0.00 | 397 | 0.67 |  | |
| Chios | 23 | 0.91 | 0.32 | 0.18 | 1.93 | 0.00 | 334 | 0.49 |  | |
| Churra | 118 | 0.96 | 0.36 | 0.11 | 1.99 | 0.00 | 600 | 0.73 |  | |
| Comisana | 24 | 0.94 | 0.36 | 0.06 | 1.97 | 0.00 | 589 | 0.62 |  | |
| Leccese | 22 | 0.95 | 0.37 | 0.12 | 1.97 | 0.01 | 556 | 0.63 |  | |
| MacarthurMerino | 10 | 0.60 | 0.22 | 0.42 | 1.61 | 0.00 | - | - |  | |
| Meat Lacaune | 76 | 0.96 | 0.37 | 0.10 | 1.99 | 0.01 | 835 | 0.74 |  | |
| Merinolandschaf | 21 | 0.93 | 0.35 | 0.11 | 1.96 | 0.00 | 407 | 0.63 |  | |
| Milk Lacaune | 100 | 0.96 | 0.36 | 0.11 | 1.99 | 0.00 | 714 | 0.73 |  | |
| Ojalada | 24 | 0.95 | 0.37 | 0.06 | 1.98 | 0.00 | 588 | 0.68 |  | |
| Rambouillet | 102 | 0.96 | 0.36 | 0.14 | 1.99 | 0.00 | 709 | 0.77 |  | |
| Rasa Aragonesa | 20 | 0.95 | 0.38 | 0.04 | 1.98 | 0.01 | 780 | 0.70 |  | |
| Sardinian Ancestral Black | 20 | 0.92 | 0.33 | 0.11 | 1.94 | 0.02 | 306 | 0.50 |  | |
|  |  |  |  |  |  |  |  |  |  | |
| **Central and Continental Europe** |  |  |  |  |  |  |  |  |  | |
| Black Headed Mutton | 24 | 0.92 | 0.33 | 0.18 | 1.95 | 0.01 | 247 | 0.51 |  | |
| Bundner Oberlander | 21 | 0.92 | 0.34 | 0.09 | 1.94 | 0.01 | 253 | 0.54 |  | |
| East Friesian Brown | 39 | 0.90 | 0.30 | 0.26 | 1.92 | 0.00 | 186 | 0.46 |  | |
| East Friesian White | 9 | 0.80 | 0.29 | 0.22 | 1.82 | 0.00 | - |  |  | |
| Engadine Red | 21 | 0.94 | 0.36 | 0.08 | 1.97 | 0.00 | 482 | 0.59 |  | |
| Swiss Black-Brown Mountain | 23 | 0.93 | 0.35 | 0.11 | 1.95 | 0.00 | 333 | 0.55 |  | |
| Swiss Mirror | 20 | 0.92 | 0.35 | 0.11 | 1.95 | 0.00 | 262 | 0.56 |  | |
| Swiss White Alpine | 21 | 0.93 | 0.35 | 0.11 | 1.96 | 0.01 | 374 | 0.56 |  | |
| Valais Black Nose | 21 | 0.87 | 0.31 | 0.24 | 1.90 | 0.00 | 288 | 0.47 |  | |
| Valais Red | 21 | 0.86 | 0.31 | 0.18 | 1.89 | 0.00 | 173 | 0.44 |  | |
|  |  |  |  |  |  |  |  |  |  | |
| **Northern Europe** |  |  |  |  |  |  |  |  |  | |
| Australian Coopworth | 19 | 0.93 | 0.36 | 0.07 | 1.96 | 0.01 | - | - |  | |
| Australian Poll Dorset | 108 | 0.95 | 0.34 | 0.15 | 1.98 | 0.01 | 318 | 0.58 |  | |
| Australian Suffolk | 109 | 0.96 | 0.37 | 0.08 | 1.99 | 0.01 | 569 | 0.68 |  | |
| Boreray | 17 | 0.77 | 0.26 | 0.28 | 1.89 | 0.00 | - | - |  | |
| Border Leicester | 48 | 0.87 | 0.29 | 0.34 | 1.79 | 0.00 | 242 | 0.45 |  | |
| Dorset Horn | 21 | 0.87 | 0.29 | 0.20 | 1.89 | 0.00 | 134 | 0.37 |  | |
| Finnsheep | 96 | 0.95 | 0.36 | 0.14 | 1.98 | 0.01 | 795 | 0.63 |  | |
| Galway | 49 | 0.93 | 0.33 | 0.15 | 1.95 | 0.00 | 322 | 0.52 |  | |
| German Texel | 43 | 0.95 | 0.35 | 0.13 | 1.97 | 0.00 | 448 | 0.57 |  | |
| Irish Suffolk | 55 | 0.93 | 0.33 | 0.22 | 1.95 | 0.02 | 300 | 0.52 |  | |
| New Zealand Romney | 21 | 0.92 | 0.35 | 0.14 | 1.95 | 0.04 | 405 | 0.52 |  | |
| New Zealand Texel | 21 | 0.92 | 0.34 | 0.14 | 1.94 | 0.02 | 282 | 0.50 |  | |
| Old Norwegian spaelsau | 15 | 0.90 | 0.35 | 0.17 | 1.94 | 0.00 | - | - |  | |
| Scottish Blackface | 56 | 0.95 | 0.36 | 0.11 | 1.98 | 0.01 | 528 | 0.61 |  | |
| Scottish Texel | 80 | 0.94 | 0.33 | 0.13 | 1.97 | 0.01 | 305 | 0.50 |  | |
| Soay | 110 | 0.78 | 0.26 | 0.33 | 1.82 | 0.00 | 194 | 0.46 |  | |
| SpaelColoured | 3 | 0.67 | 0.28 | 0.18 | 1.71 | 0.00 | - | - |  | |
| Spael White | 29 | 0.90 | 0.33 | 0.19 | 1.93 | 0.00 | 339 | 0.49 |  | |
| Wiltshire | 23 | 0.80 | 0.26 | 0.33 | 1.82 | 0.03 | 100 | 0.37 |  | |
|  |  |  |  |  |  |  |  |  |  | |
|  |  |  |  |  |  |  |  |  | |  |

Genetic diversity indices measured within breed. **n** gives the number of individuals used to calculate the proportion of SNP displaying polymorphism (***P*_n_**); expected heterozygosity or gene diversity (***H*_e_**); the inbreeding coefficient (**F**); allelic richness (***A*_r_**) and private allele richness (**p*A*_r_**). The effective population size (***N*_e_**) was calculated using linkage disequilibrium for breeds with at least 20 genotyped individuals as described in [21]. ***HS*_MER_** gives the haplotype sharing, at 25 – 50 kb, between Merino and other breeds plotted in Figure 1C. Values approaching one indicate increasing haplotype sharing with Merino as described in Figure S6.
