# Supplementary material for: Genome-Wide Analysis of the World's Sheep Breeds Reveals High Levels of Historic Mixture and Strong Recent Selection
Source: PLoS Biol. 2012 Feb 7;10(2):e1001258. doi: 10.1371/journal.pbio.1001258 (PMC3274507; doi:10.1371/journal.pbio.1001258)
Supplement: Table S5 — Enrichment analysis of gene ontology (GO) terms for genes in regions under selection. (DOC) [file pbio.1001258.s016.doc]

**Table S5. Enrichment analysis of gene ontology (GO) terms for genes in regions under selection.**

|  |  |
| --- | --- |
|  |  |
| **Biological Process** | **P-value ^1^** |
|  |  |
|  |  |
| Regulation of bone resorption | 4.00E-05 |
| Regulation of bone remodeling | 5.53E-05 |
| Glycosphingolipid metabolic process | 1.27E-04 |
| Protein ubiquitination involved in ubiquitin-dependent protein catabolic process | 1.61E-04 |
| Glycolipid metabolic process | 2.48E-04 |
| Regulation of tissue remodeling | 2.48E-04 |
| Regulation of collateral sprouting of intact axon in response to injury | 2.54E-04 |
| Multicellular organismal development | 6.13E-04 |
| Dendritic cell chemotaxis | 7.53E-04 |
|  |  |
|  |  |

^1^ The enrichment analysis sought to identify gene ontology (GO) terms that were significantly overrepresented in 181 genes residing within the 31 regions under selection in the sheep genome (Table 1). The terms associated with the 181 genes were compared against a background set of 11,098 genes. Each of the 11,098 genes contain a SNP present on the *ovine* SNP50 Beadchip, or a SNP within 2.5 Kb. Comparison of the two gene lists (target and background) was performed using the software GOrilla, which implements a hypergeometric distribution and mHG p-value approach to determine significance [58].
