# Supplementary material for: Genome-Wide Analysis of the World's Sheep Breeds Reveals High Levels of Historic Mixture and Strong Recent Selection
Source: PLoS Biol. 2012 Feb 7;10(2):e1001258. doi: 10.1371/journal.pbio.1001258 (PMC3274507; doi:10.1371/journal.pbio.1001258)
Supplement: Table S6 — Selection signals identified in both sheep and cattle. (DOC) [file pbio.1001258.s017.doc]

**Table S6. Selection Signals Identified in Both Sheep and Cattle.**

|  |  |  |  |  |  |
| --- | --- | --- | --- | --- | --- |
| **Region** | **Sheep Chr: Mb** | **Cow Chr: Mb** | **Animals** | **Study** | **Common Genes Between Studies** |
|  |  |  |  |  |  |
| 3 | 2: 246.3-246.4 | 2: 117.7-129.0 | African cattle | [37] | DIS3L2, LOC100125266,ALPI, LOC528262, LOC516378, CHRND, **CHRNG**, LOC781731, EIF4E2 |
| 4 | 3: 141.4-141.5 | 5: 26.8-31.0 | African cattle | [37] | **HOXC4**, MIR615, **HOXC5**, **HOXC6**, MIR196A-2, **HOXC11**, **HOXC12**, **HOXC13** |
| 5 | 3: 164.5-165.4 | 5: 47.8-54.3 | African cattle | [37] | MIR763, MSRB3, LEMD3, MIR2429, WIF1 |
|  |  | 5: 52.6-53.0 | Cattle HapMap | [15] | WIF1 |
| 7 | 6 :40.9-41.7 | 6: 37.4-38.7 | Dairy cattle | [35] | SPP1, IBSP, LAP3, MED28, LOC509580, FAM184B, DCAF16, NCAPG, |
| 8 | 6: 76.7-76.7 | 6: 72.5-73.7 | Creole cattle | [24] | **KIT** |
| 9 | 6: 103.2-103.7 | 6: 89.3-105.4 | African cattle | [37] | ANTXR2, **FGF5**, PRDM8 |
| 14 | 10: 29.0-30.0 | 12: 28.4-29.5 | Creole cattle | [24] | **RXFP2** |
|  |  | 12: 20.6-32.6 | African cattle | [37] | FRY, LOC788183, B3GALTL, **RXFP2** |
|  |  | 12: 25.8-26.1 | Cattle HapMap | [15] |  |
| 15 | 10: 30.5-30.8 | 12: 20.6-32.6 | African cattle | [37] | USPL1, KATNAL1 |
| 26 | 19: 33.2-33.3 | 22: 32.3-38.5 | African cattle | [37] | **MITF** |
| 30 | 25: 7.1-7.3 | 28: 5.3-6.4 | Dairy cattle | [38] | TARBP1, IRF2BP2 |
| 31 | 25: 28.8-29.1 | 28: 26.8-27.3 | Dairy cattle | [38] | **CDH23**, C28H10orf54 |
|  |  |  |  |  |  |
|  |  |  |  |  |  |

31 regions spanning 17.8 Mb containing outlier SNP (this study) were compared against the results from eight experiments reporting positive selection in cattle [15,24,35-40]. The genomic coordinates from 215 regions of the cattle genome (Btau4.0) were taken from the eight published studies, migrated onto the sheep genome (OARv1.0) and compared with the results in Table 1. The 215 regions spanned a total of 379 Mb, or around 12% of the cattle genome and have an average size of 2.04 Mb. A total of 11 out of 31 regions were identified in common between this study and the cattle experiment indicated. The genomic location and gene content of regions independently identified in multiple studies are given. Symbols in bold indicate genes that have prior evidence for a role in pigmentation, body size, horn morphology, reproduction or domestication.
